# Making and Breaking of Gels: Stimuli-Responsive Properties of Bis(Pyridyl-*N*-oxide Urea) Gelators

**DOI:** 10.3390/molecules26216420

**Published:** 2021-10-24

**Authors:** Sreejith Sudhakaran Jayabhavan, Dipankar Ghosh, Krishna K. Damodaran

**Affiliations:** Department of Chemistry, Science Institute, University of Iceland, Dunhagi 3, 107 Reykjavík, Iceland; ssj37@hi.is (S.S.J.); dipankar@hi.is (D.G.)

**Keywords:** LMWGs, stimuli-responsive, structural modification, pyridyl urea, pyridyl-*N*-oxide

## Abstract

The structural modification of existing supramolecular architecture is an efficient strategy to design and synthesize supramolecular gels with tunable and predictable properties. In this work, we have modified bis(pyridyl urea) compounds with different linkers, namely hexylene and butylene, to their corresponding bis(pyridyl-*N*-oxide urea). The gelation properties of both the parent and the modified compounds were studied, and the results indicated that modification of the 3-pyridyl moieties to the corresponding 3-pyridyl-*N*-oxides induced hydrogelation. The stability of the parent and modified compounds were evaluated by sol-gel transition temperature (*T_gel_*) and rheological measurements, and single-crystal X-ray diffraction was used to analyze the solid-state interactions of the gelators. The morphologies of the dried gels were analyzed by scanning electron microscopy (SEM), which revealed that the structural modification did not induce any prominent effect on the gel morphology. The stimuli-responsive behavior of these gels in the presence of salts in DMSO/water was evaluated by rheological experiments, which indicated that the modified compounds displayed enhanced gel strength in most cases. However, the gel network collapsed in the presence of the chloride salts of aluminum(III), zinc(II), copper(II), and cadmium(II). The mechanical strength of the parent gels decreased in the presence of salts, indicating that the structural modification resulted in robust gels in most cases. The modified compounds formed gels below minimum gel concentration in the presence of various salts, indicating salt-induced gelation. These results show the making and breaking ability of the gel network in the presence of external stimuli (salts), which explains the potential of using LMWGs based on *N*-oxide moieties as stimuli-responsive materials.

## 1. Introduction

Stimuli-responsive low molecular weight gelators (LMWGs) [1,2,3,4,5] are an excellent class of soft materials because the gelation properties can be switched on/off by an external stimulus, such as heat, light, sound, redox, pH, and salts/ions. These semi-solid materials with unique physical properties display various applications [6,7,8,9,10,11,12,13] in catalysis, cell culture, crystal growth media, drug delivery, tissue engineering, and sensing and dynamic gels. The supramolecular architecture of LMWGs (3-D network) is fabricated by the molecular self-assembly of the gelator with entrapped solvent molecules, which is stabilized by various non-bonding interactions [12,14,15,16,17,18], such as hydrogen bonding, van der Waals interactions, π-π stacking, etc. Various analytical techniques (spectroscopic, microscopic, and X-ray diffraction) [19,20,21,22,23] have been used to study the mechanism of gelation, and X-ray diffraction methods have been proven to be one of the promising tools in understanding the gelator structure and its aggregation behavior [24,25,26,27,28]. However, predicting the gel structure or controlling the gel state properties is difficult because of the dynamic nature of the non-bonding interactions, leading to low molecular order in the overall gel state [12,14,15,16,17,18,29]. Thus, understanding the molecular self-assembly of supramolecular gels is a challenging task, enabling us to design LMWGs with predictable properties. The role of specific interactions in gel formation can be analyzed by modifying the functional groups of LMWGs that play an important role in gel network formation.

The modification of these functional groups will lead to the alteration of specific interactions arising from the particular functional group, which can be considered as an excellent strategy to evaluate the role of specific interactions in gel formation [30,31]. For example, Zinchuan et al. have synthesized a cholesterol-based dendrimer gelator from the parent terminal alkyne-based gelator and have shown that the modified compounds formed gel in polar solvents compared to the parent compound, which only formed gel in nonpolar solvents [32]. McNeil’s group has developed nitrite sensor-based LMWGs from a known azosulfonate gelator scaffold [33]. We have shown that the tris(pyridyl-*N*-oxide) gelator obtained by modifying trimesic amide-based LMWG can be used as a crystallizing medium for copper(II) isonicotinate–*N*-oxide complex [34]. The gelation properties of the parent and the modified gelators were compared to analyze the importance of specific interactions and the nature of the functional groups in tuning gelation properties by modifying pyridyl groups of *N*–(4–pyridyl)isonicotinamide to *N*–oxide groups [31]. The compounds with *N*–oxide groups have several advantages over the parent pyridyl-*N* atom due to the presence of additional lone pairs in the *N*-oxide moiety, which could lead to enhanced interactions with solvent/guest molecules. For example, Xu et al. showed that modified metal-organic frameworks with *N*-oxide moieties displayed enhanced C_2_H_2_ and CO_2_ adsorptions [35]. Compounds with pyridine-*N*-oxide moieties are an excellent organocatalyst in various racemic and enantioselective reactions and act as oxidant in various oxidation reactions [36]. We have also modified pyridyl mono(urea)-based compounds [25] to pyridyl-*N*-oxide mono(urea) compounds and showed that the modified compounds formed highly resistant gels towards inorganic salts/ions [30]. We have successfully correlated the solid-state interactions with the gelation properties in mono(urea) LMWGs [30], which prompted us to evaluate the effect of structural modification in bis(pyridyl urea)-based compounds.

The gelation properties of bis(pyridyl urea) compounds have extensively been studied due to their ease of synthesis, cost-effectiveness, flexibility towards structural modification, and stimuli-responsive properties [29,37,38,39]. The presence of pyridyl groups could enhance the stimuli-responsive properties of bis(pyridyl urea) LMWGs in the presence of salts/ions [38]. The addition of salts could alter the non-bonding interactions, which might disrupt the self-assembly of the gelator constructively, by triggering gelation [40,41,42], or destructively, leading to gel dissolution [4,5]. The electrostatic interaction associated with the ions and the acidic/basic properties of the cations/anions plays a significant role in the interaction between the gelator and the salt [5,43]. Furthermore, the functional groups in a gelator also play a vital role, which acts as a binding or reaction site towards salts/ions [43,44]. Yang et al. reported the fluoride ion-induced gel-sol transition in a naphthalene-based bis(urea) gelator and have shown that the process can be reversed by adding trifluoroacetic acid [45]. The detection of iodide ions by bis(pyridyl urea) gels in the presence of various other anions was reported by Ghosh et al. [46]. Piepenbrock et al. reported the silver salt-induced gelation of bis(pyridyl urea) in tetrahydrofuran–water mixtures [47]. Thus, LMWGs based on bis(pyridyl urea) are excellent stimuli-responsive soft materials. In this work, we will evaluate the stimuli-responsive properties of bis(pyridyl urea) and the modified compounds in the presence of various cations and anions.

## 2. Results and Discussion

### 2.1. Design and Synthesis

**Scheme 1:**
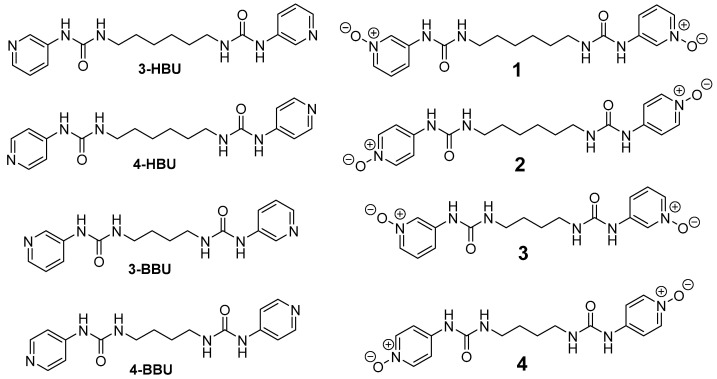
Parent bis(pyridyl urea) and the corresponding *N*–oxide compounds obtained by modifying the pyridyl moieties.

The modification of the pyridyl functionality in pyridyl urea-based compounds could alter the N—H⋯N interactions to N—H⋯O interactions. Recently, we have shown that modifying pyridyl groups to the corresponding pyridyl-*N*-oxides [30] can restore the N—H⋯O interactions, leading to enhanced gelation ability. In this work, we have selected bis(pyridyl urea)-based compounds (**3-HBU [29]**, **3-BBU** [48], **4-HBU**, and **4-BBU**; see Scheme 1) as the parent compounds. The structural modification of these compounds will enable us to study the effect of functional group modification on the gelation properties of bis(pyridyl urea) systems and the specific role of N—H⋯O/N—H⋯N synthons in gel formation. The linker in bis(urea) compounds also plays an important role in gel formation by influencing the spatial arrangements of the functional groups and tuning the hydrophilic/hydrophobic interactions [8,49]. Thus, we have selected hexylene and butylene-based linkers connected to the 3-pyridyl/4-pyridyl urea moieties (Scheme 1). The hexylene linked bis-ureas (**3-HBU** and **4-HBU**) were synthesized by reacting 3–aminopyridine/4–aminopyridine with hexamethylene diisocyanate (Scheme S1) [29]. The butylene analogs (**3-BBU** and **4-BBU**) were obtained from the reaction of 1,4-diaminobutane and 3-pyridyl isocyanate/4-pyridyl isocyanate (Scheme S2). The oxidation of the pyridyl groups to pyridyl-*N*-oxide by 3–chloroperoxybenzoic acid (Scheme S3) resulted in compounds **1**–**4** (Scheme 1).

### 2.2. Gelation Experiments

The gelation studies of the parent bis(pyridyl urea) compounds and the modified *N*-oxides were performed in various solvents/solvent mixtures. A standard procedure was followed for gelation experiments, 1.0 mL of the corresponding solvent was added to 10.0 mg of the compound in a standard 7.0 mL sealed vial, and the mixture was heated to obtain a clear solution, which was cooled to room temperature and left undisturbed for 24.0 h. Gelation was not observed for all the compounds at this concentration, which prompted us to perform the gelation test at a higher concentration (up to 5.0 *wt*/*v*%; Table S1). A vial inversion test was performed to confirm the gelation, and the gelation test indicated that both the parent and modified compounds were insoluble in hydrocarbons, such as toluene, xylenes, and mesitylene, and polar aliphatic solvents (THF, 1,2-dibromoethane, and acetonitrile). The modified *N*-oxide compounds were found to be less soluble in organic solvents but more soluble in water, which can be attributed to the strong hydrophilic interactions of the *N*-oxide moiety.

**Figure 1:**
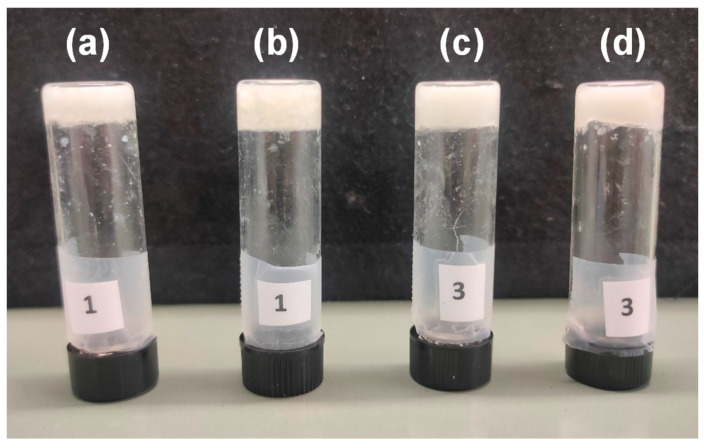
Gels obtained with compound **1** in water (**a**) and DMSO/water (1:1 *v*/*v*) (**b**), and compound **3** in water (**c**) and DMSO/water (1:1 *v*/*v*) (**d**).

The parent bis(pyridyl urea) compounds were insoluble in water, but the modified compounds **1**, **3**, and **4** formed hydrogels at 2.0, 3.0, and 7.0 wt%, respectively, indicating that the structural modification has induced hydrogelation (Figure 1). We have also analyzed the gelation properties in mixed aqueous solvents (Table S1), and compounds **3-HBU**, **3-BBU**, and **3** formed gels in DMF/water (1:1, *v*/*v*). Gelation was also observed in a DMSO/water (1:1, *v*/*v*) and ethylene glycol (EG)/water 3:7 (*v*/*v*) mixture for **3-HBU**, **3-BBU**, **1**, and **3**. However, gelation was not observed for the 4-pyridyl derivatives (**4-HBU** and **4-BBU**) and **2** in the above solvents/solvent mixtures (Table S1), indicating the importance of relative positions of the functionalities in gel formation [24,37].

The relative gelling ability and thermal stability of the parent and modified gels were analyzed by comparing the minimum gel concentration (MGC) and sol-gel transition temperature (*T_gel_*) experiments, respectively. MGC is the minimum concentration of the compound, which is required to form a stable gel under ambient conditions. We have chosen DMSO/water (1:1, *v*/*v*) and EG/water (3:7, *v*/*v*) as the solvent systems since both parent (**3-HBU** and **3-BBU**) and modified compounds (**1** and **3**) formed gels in these solvent mixtures. The MGC of compound **1** was found to be 3.8 *wt*/*v*% in DMSO/water (1:1, *v*/*v*), which is slightly less than the MGC of **3-HBU** (4.0 *wt*/*v*%) (Table S2). Compound **3** formed gel at a lower concentration compared to **3-BBU** in all cases. Thus, the functional group modification led to a decrease in MGC of the pyridyl-*N*-oxide compounds. We have also analyzed the effect of the hexylene and butylene linkers, which indicated that increasing the alkyl chain length resulted in lower MGC (Table S2).

### 2.3. Thermal Stability

The gel-to-sol transition temperature (*T_gel_*) was recorded to compare the thermal stability of the modified gelators with the parent compounds. The thermal stability of **3-HBU** was found to be very high in comparison with gelator **1** in both DMSO/water (1:1, *v*/*v*) and EG/water (3:7, *v*/*v*). A similar trend was observed for **3-BBU** in comparison with compound **3**, where the *T_gel_* value of **3-BBU** was found to be considerably higher (Table 1). Thus, the modification of functional groups could alter the thermal stability, which indicated that the nature of the functional groups played an important role in determining the thermal stability of the gel network. The thermal stability of LMWGs also depends on the linkers, and we have compared the *T_gel_* of hexylene and butylene-based compounds. These results indicated that the hexylene-based **3-HBU** gel was more thermally stable than the butylene-based **3-BBU** in 1:1 (*v*/*v*) DMSO/water. A similar trend was observed for the modified compounds (**1** and **3**) in DMSO/water (1:1, *v*/*v*), but a reverse trend was observed in EG/water (3:7, *v*/*v*) (Table 1), indicating that both linkers and solvents played a vital role in dictating the thermal stability of LMWGs.

### 2.4. Rheology

The mechanical and solid-like properties of the bis(pyridyl-*N*-oxide urea) gels and the corresponding parent gelators were evaluated using rheology [50,51]. The mechanical strengths of **3-HBU** and gelator **1** were measured at 5.0 *wt*/*v*% in DMSO/water (1:1, *v*/*v*) and EG/water (3:7, *v*/*v*). Initially, an oscillatory strain-sweep experiment to determine the linear viscoelastic region (LVR) was performed to check whether the characteristic of the gel is retained or not because the gel networks undergo reversible deformation within the LVR. The results indicated that both the modified gelator **1** and its parent **3-HBU** had narrow LVR as the storage modulus G’ decreased after 0.05% of the shear strain (Figure S1). The point at which the elastic gel converts to a viscous fluid, accompanied by a sudden decrease in the G’, is known as the crossover point [50,51].

The crossover points for **3-HBU** and **1** in both the solvent mixtures were found to be within 3.0–10.0% of shear strain. Similar experiments were also performed to compare the gel strength of **3-BBU** and **3** in DMSO/water (1:1, *v*/*v*) and EG/water (3:7, *v*/*v*) at 5.0 *wt*/*v*%, respectively. Analyzing the LVR indicated that for **3-BBU,** the G’ decreased on increasing the shear strain above 0.02% but decreased after 0.05% of the shear strain for gelator **3**. The crossover point for **3-BBU** gelator was found to be around 0.5–2.0% of strain, but this was slightly higher (3.0–10.0)% of strain for gelator **3** (Figure S2). These results demonstrated that the mechanical strength of the parent and modified compounds in DMSO/water (1:1, *v*/*v*) and EG/water (3:7, *v*/*v*) displayed a narrow LVR in all cases, which proved that the gelators formed soft gels. Furthermore, above 10.0% of strain, all the gels were found to behave as a viscous fluid, indicated by the steep decrease in the G’.

**Figure 2:**
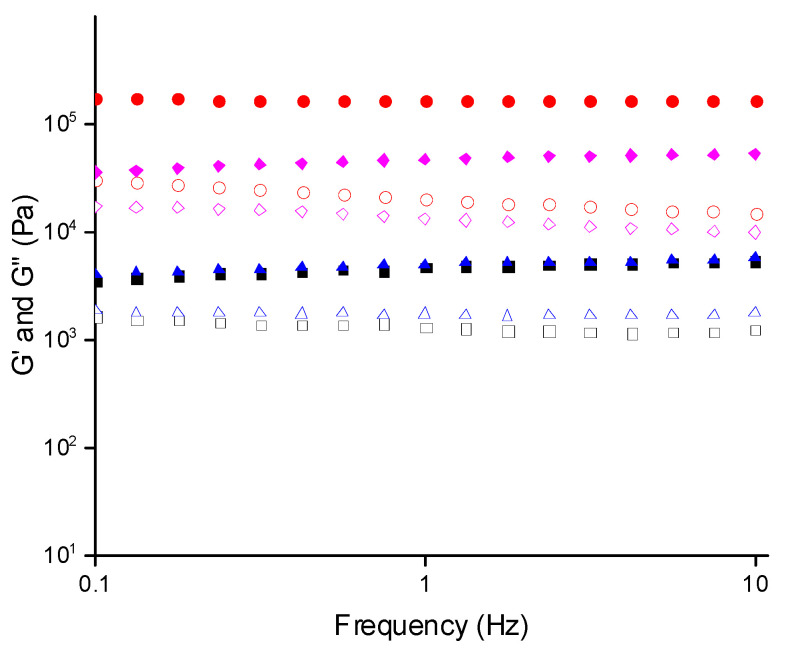
Frequency sweep experiments of gels of **3-BBU** and **3**, 5.0 *wt*/*v*% at 25.0 °C with a constant strain of 0.02%. Color codes: In EG/water (3:7, *v*/*v*) **3-BBU** G′ (■) and G′′ (□), compound **3** G′ (●) and G′′ (○), and in DMSO/water (1:1, *v*/*v*) **3-BBU** G′ (▲) and G′′ (Δ), and compound **3** G′ (♦) and G′′ (◊).

Frequency sweep experiments were performed at a constant strain of 0.02% (within LVR) in a range of 0.1–10.0 Hz, which displayed a constant elastic (*G*′) and viscous (*G*″) moduli under varying frequency. The relative gel strength of **3–HBU** and **1** was compared by performing frequency sweep experiments on the gels prepared at 5.0 *wt*/*v*% in EG/water (3:7, *v*/*v*). The modified gelator **1** displayed a higher elastic modulus (G’) than the parent gelator **3-HBU** (~25 times). In DMSO/water (1:1, *v*/*v*), the G’ for **1** at 5.0 *wt*/*v*% was around ten times higher than the gelator **3-HBU** (Figure S3). The experiments performed with **3-BBU** and gelator **3** at 5.0 *wt*/*v*% also displayed similar results. Similarly, the G’ for gelator **3** was found to be approximately 25 times higher than **3-BBU** in EG/water (3:7, *v*/*v*) and around ten times higher in DMSO/water (1:1, *v*/*v*) (Figure 2). These results showed that the functional group modification enhanced mechanical strength, presumably due to the alteration of non-bonding interactions in the modified compounds.

### 2.5. Gel Morphology

**Figure 3:**
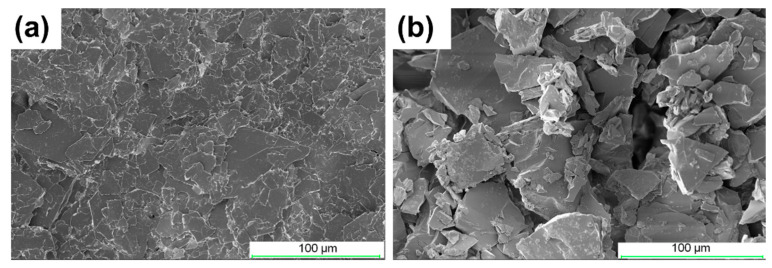
SEM images of xerogels of (**a**) **3-HBU,** and (**b**) **1** in DMSO/water (1:1, *v*/*v*) at 4.0 *wt*/*v*%, respectively.

The surface morphology of the xerogels was analyzed by scanning electron microscopy (SEM). We have performed SEM of the xerogels from DMSO/water (1:1, *v*/*v*) to compare the morphology of the xerogels of gelator **1** and **3-HBU**. Xerogel of **3-HBU** prepared at 4.0 *wt*/*v*% in DMSO/water (1:1, *v*/*v*) displayed plate-like morphology with dimensions ranging from 5.0–40.0 μm (Figure 3a), and the structurally modified gelator **1** also displayed fibers (10.0–60.0 μm) with similar plate-like morphology (Figure 3b). Similarly, the xerogels of the parent gelator **3-BBU** (dimension 20.0–100.0 μm) and **3** (dimension 2.0–10.0 μm) in DMSO/water (1:1, *v*/*v*) at 5.0 *wt*/*v*% displayed plate-shaped morphology (Figure S4a,b).

**Figure 4:**
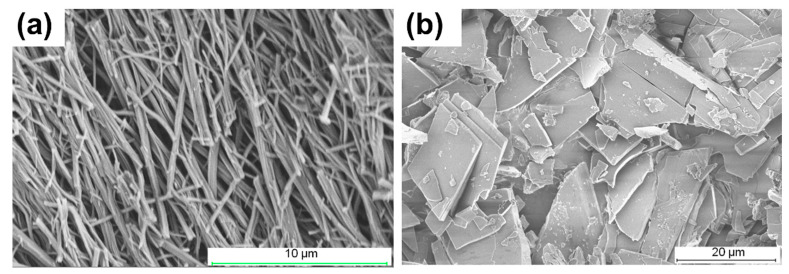
SEM images of xerogels in water at 3.0 wt%: (**a**) **1** and (**b**) **3**.

We have also performed the SEM of the xerogels of **1** and **3** in water at 3.0 wt%, and the hydrogelator **1** displayed fibrous morphology with thin and long fibers of thickness ranging from 0.1–0.5 μm (Figure 4a), but a plate-shaped morphology with a dimension of 2.0–16.0 μm was observed for gelator **3** (Figure 4b). Thus, the functional group modification of the bis(pyridyl urea)-based compounds to the corresponding bis(pyridyl-*N*-oxide urea) did not affect the morphologies of the gelators in DMSO/water (1:1, *v*/*v*), but solvent-induced morphological change was observed in hydrogels of **1,** indicating the role of solvents in the morphology of the gel fibers.

### 2.6. Single Crystal X-ray Diffraction

Single crystal X-ray diffraction analysis will enable us to analyze the non-bonding interactions of the *N*-oxide gelators in solid-state, which could be correlated to the gelation properties. Needle-shaped crystals of gelator **1** were obtained from DMSO/water, and single-crystal X-ray analysis revealed that compound **1** crystallized in monoclinic space group C2/c with one water molecule (**1**·H_2_O) (Table S3).

**Figure 5:**
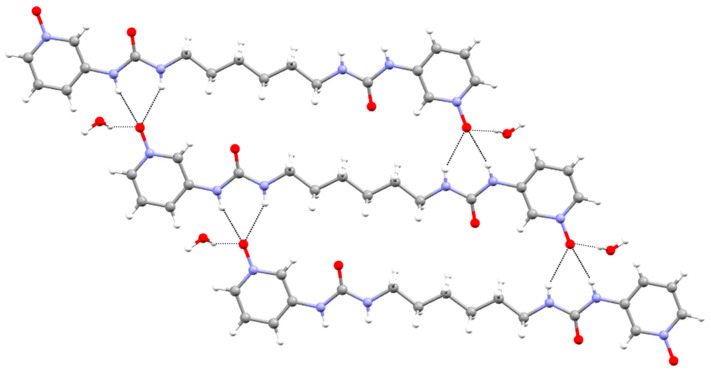
One-dimensional chain of **1**·H_2_O displaying bifurcated hydrogen bonding.

Compound **1** has an inversion center resulting in the anti-conformation of the urea and the *N*-oxide moieties. The urea moieties displayed bifurcated hydrogen bonding with the *N*-oxide moieties via N—H⋯O interactions (2.8749(16) and 2.9531(17) Å; Table S4), resulting in a one-dimensional urea α-tape-like architecture (Figure 5) similar to complementary urea N—H⋯O=C aggregation. The one-dimensional chains are interconnected by O—H⋯O interactions between the pyridyl-*N*-oxide moiety and the solvent water molecule (Figure S5a).

**Figure 6:**
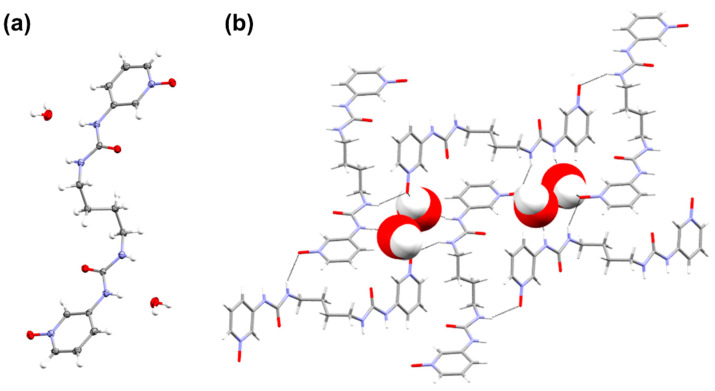
(**a**) Molecular structure of **3**·2H_2_O and (**b**) two-dimensional network with entrapped water molecule (space fill model).

Gelator **3** was crystallized with two water molecules (**3**·2H_2_O) in a monoclinic space group P2_1_/n (Table S3). The overall conformation of the molecule was non-planar, and the urea and the *N*-oxide moieties adopted an anti-confirmation due to the inversion center (Figure 6a). One of the nitrogen atoms of the urea moieties was hydrogen-bonded to the pyridyl-*N*-oxide via N—H⋯O interactions (3.0246(16) Å) to form a two-dimensional hydrogen bonded network. The solvent water molecule was entrapped in this network (Figure 6b), which was stabilized by hydrogen bonding interactions with the other nitrogen atom of the urea motif (N—H⋯ O = 2.8011(17) Å) and the oxygen atom of the pyridyl-*N*-oxide moieties of adjacent network (O⋯H—O = 2.8329(18) and 2.9013(18) Å) to form a porous architecture (Figure S5b), which was further stabilized by various non-bonding interactions (Table S4).

The compound **4-BBU** was crystallized in the monoclinic space group P2_1_/n with an inversion center. The urea moieties displayed anti-confirmation and the molecular structure was non-planar similar to **3** (Figure S6a). One of the nitrogen atoms of the urea moieties of **4-BBU** displayed N—H⋯O interactions (2.9871(15) Å) with the oxygen atom of the carbonyl moieties to form a one-dimensional hydrogen-bonded chain (Figure S6b). The second nitrogen atom of the urea moieties interacted with the pyridyl nitrogen from the adjacent molecule via N⋯H—N interactions (2.986(2) Å), which connected the two orthogonal one-dimensional chains to form a two-dimensional hydrogen-bonded network. The corresponding *N*-oxide compound **4** also crystallized in monoclinic space group P2_1_/n with an inversion center, and the urea moieties adopted an anti-confirmation (Figure S7a). The nitrogen atoms of the urea moieties displayed bifurcated hydrogen bonding with the oxygen atom of the pyridyl-*N*-oxide moieties (2.7887(18) and 2.9988(18) Å) to form a two-dimensional hydrogen-bonded sheet architecture (Figure S7b) with cavities. The solvent water molecules located in these cavities (Figure S7c) were hydrogen-bonded to the oxygen atoms of the carbonyl (2.889(2) Å) and pyridyl-*N*-oxide (2.788(2) Å) moieties, respectively.

The crystal structures of the parent compounds were compared to the modified compounds to analyze how structural modification affected the non-bonding interactions in the solid-state. The comparison of the solid-state structures of **3-BBU** [48] and the corresponding *N*-oxide **3** indicated that the structural modification changed the hydrogen bonding pattern of the urea moieties of **3-BBU** from N⋯H—N/N—H⋯O interactions [48] to N—H⋯O interactions in **3**. The comparison of structures of **4-BBU** and the corresponding *N*-oxide **4** also revealed the alteration of non-bonding interactions; for example, the N⋯H—N interactions were also replaced by N—H⋯O interactions in **4**. We were not able to compare the solid-state structure of **1** with the parent compound because crystallization **3-HBU** was unsuccessful, despite several trials. We have compared the solid-state interactions of gelator **1** and **3**, and gelator **1** displayed a urea α-tape-like architecture via N—H⋯O interactions involving the urea and pyridyl-*N*-oxide moieties similar to complementary urea hydrogen bonding. However, the N—H⋯O interactions in **3** resulted in a two-dimensional hydrogen-bonded network, indicating that linkers play an important role in dictating the one-dimensional chain, which is crucial for better gelation properties [48].

### 2.7. X-ray Powder Diffraction (XRPD)

X-ray Powder Diffraction is an important technique to identify the phase purity of bulk solids, which is performed by comparing the XRPD pattern of the bulk crystals with the simulated pattern obtained from the crystal structure. Moreover, the comparison of the powder X-ray patterns of the dried gels with the simulated pattern obtained from the crystal structure may provide useful information about the interactions involved in the gel network formation [14,19,28,31,52,53,54]. Although the drying process of the gels can lead to artifacts, this method can be considered as an elegant approach to correlate the self-assembly process in LMWGs [55].

**Figure 7:**
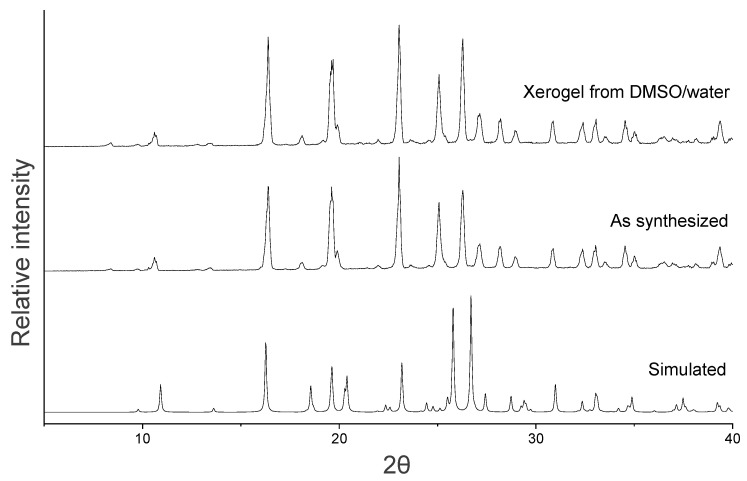
Comparison of the simulated pattern from single-crystal data, bulk-crystals synthesized from DMSO/water, and xerogel from DMSO/water (1:1, *v*/*v*) of compound **3**.

We have compared the XRPD pattern of the xerogel of **1** obtained from DMSO/water (1:1, *v*/*v*) at 4.0 *wt*/*v*%, which did match with the simulated pattern. The XRPD pattern of the bulk crystals of gelator **1**·H_2_O was also found to be similar to the simulated pattern, except an extra peak at 2θ ≈ 11.0°, presumably due to the solvent water molecules (Figure S8). Similarly, for gelator **3**, the XRPD pattern of the bulk crystals and the xerogel obtained from DMSO/water (1:1, *v*/*v*) at 5.0 *wt*/*v*% matched with the simulated pattern of the single crystal of **3**·2H_2_O (Figure 7)**.** However, a slight shift was observed with bulk crystals and xerogels at higher 2θ (> 25.0°) compared to the simulated pattern of the single crystal of **3**·2H_2_O. The XRPD pattern of the xerogel of **1** and **3** at 3.0 wt% in water was different from that of the simulated patterns, indicating that the gelator might adopt different structures depending on the gelling solvent (Figures S8 and S9).

We have also recorded the XRPD pattern of the **3-BBU** bulk crystals and xerogel. The XRPD patterns of the bulk crystals and the xerogels obtained from DMSO/water (1:1, *v*/*v*) at 5.0 *wt*/*v*% were superimposable, but it was found to be different from the simulated pattern (Figure S10). The XRPD pattern of **4-BBU** (as-synthesized) was identical to the simulated pattern (Figure S11), but the pattern of **4** (as-synthesized) did not match with the simulated pattern, presumably due to a different crystal packing in the presence of the solvent water molecule (Figure S12).

### 2.8. Physical Properties in the Presence of Salts

The stimuli-responsive properties of LMWGs [1,2,3,4,5,56] were studied in the presence salts/ions, for example, making or breaking of the gel network. Xu et al. have synthesized an organogel with pyrene fluorophore and urea-sulfonamide anion binding sites to show the effect of ion′s size in gelation [57]. Pang et al. reported the gel-sol transition in the presence of selective anion [58]. Adam’s group has demonstrated a Ca^2+^ induced hydrogelation at alkaline pH in LMWGs based on dipeptide derivatives [59]. The presence of pyridyl/pyridyl-*N*-oxide moieties in our compounds makes them ideal candidates as stimuli-responsive soft materials because these groups are known to interact with the salts/ions. We have previously reported the anion sensing studies of pyridyl-*N*-oxide compounds [30] and have shown that gel-sol transition can be used to detect cyanide ions [60]. This prompted us to explore the effect of salts in the gelation properties of bis(pyridyl urea) and its corresponding bis(pyridyl-*N*-oxide urea) compounds.

The effect of salts on the thermal stability and mechanical strength of the gel network was analyzed by *T_gel_* experiments and rheology, respectively. The stimuli-responsive properties were analyzed by treating the gels with various potassium salts, such as KF, KCl, KBr, KI, and KCN, in DMSO/water mixture, and the gelation properties of the modified *N*-oxide compounds were compared to the corresponding parent gelators. The gels of compounds **1** and **3-HBU** were prepared in DMSO/water (1:1, *v*/*v*) at 4.0 *wt*/*v*%, and the parent and modified gelators were stable in the presence of all the salts (1.0 and 3.0 equivalents). The thermal stability of the parent and modified compounds in the presence of these salts indicated a slightly increase in *T_gel_* values for gelator **1** in the presence of the salts (1.0 equivalents), but the values were comparable with the pure gelator for **1** at 3.0 equivalents of salts. However, the *T_gel_* values for **3** in the presence of 1.0 and 3.0 equivalents of salts were similar to the pure gelators (Table S5). The thermal stability of **3-HBU** and **3-BBU** decreased in the presence of the salts, and these results indicated that salts could interfere with gelator-solvent molecule interactions, thereby affecting the thermal stability of these gels [61].

**Figure 8:**
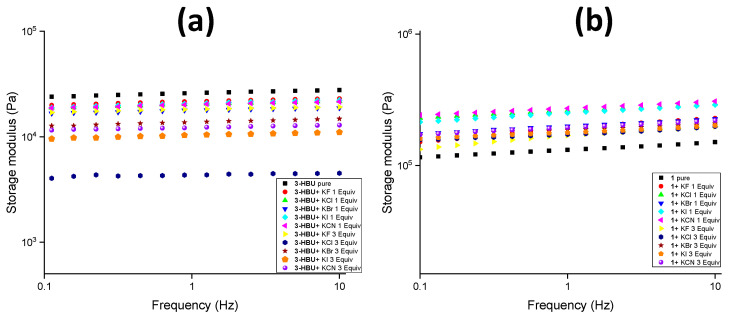
Frequency sweep experiments at 4.0 *wt*/*v*% in DMSO/water (1:1, *v*/*v*) in the presence of various salts of potassium at 25.0 °C with a constant strain of 0.02%, (**a**) **3-HBU** and (**b**) Gelator **1**.

We have analyzed the mechanical strength of the gels in the presence of salts, and the frequency sweep experiments revealed that the relative gel strength of the parent gelator **3-HBU** decreased in the presence of 1.0 and 3.0 equivalents of KF, KCl, KBr, KI, and KCN (Figure 8a). This is presumably due to the interaction of the pyridyl group with the salts affecting the non-bonding interactions responsible for the mechanical strength of the gelator. There was a considerable decrease in the mechanical strength of parent gelator **3-HBU** in the presence of 3.0 equivalents of KCl, KI, and KCN. However, a reverse trend was observed for the modified compound **1** in the presence of (1.0 and 3.0 equivalents) potassium salts (halides and cyanide). The mechanical strength was slightly reduced when the concentration of the salts was increased (3.0 equivalents) compared to the 1.0 equivalent of the salts, but the mechanical strength was higher than the pure gel in all cases (Figure 8b).

The treatment of various anions of the potassium salts (halides and cyanides) gave similar enhancement in the mechanical strength of the modified compound **1**, which indicates that the enhanced mechanical strength does not depend on the size and nature of the anions. This prompted us to check the stimuli-responsive properties of gelators in the presence of various cations, and we have selected the chloride salts of cations of group IA (sodium), group IIA (calcium and magnesium), group III (aluminum), and transition metals (copper, zinc, and cadmium; Table S6). The results indicated that enhanced mechanical properties were observed in the presence of 1.0 and 3.0 equivalents of chlorides salts of sodium, magnesium, and calcium (Figure S13a). However, weak gels were obtained at 1.0 equivalent of CuCl_2_ and CdCl_2_ (1.0 and 3.0 equivalents), but the gelation was ceased in the presence of 3.0 equivalents of CuCl_2_, 1.0 and 3.0 equivalents of ZnCl_2_ and AlCl_3_, respectively (Figure S14). Similar experiments performed with parent **3-HBU** revealed that the presence of chlorides salts (1.0 and 3.0 equivalents) of sodium, magnesium, and calcium decreased the mechanical strength compared to the pure gelator, but a reverse trend was observed for CuCl_2_ (1.0 and 3.0 equivalents) and ZnCl_2_ (1.0 equivalents), presumably due to the formation of metallogels (Figure S13b). However, the gel network collapsed in the presence of CdCl_2_ and AlCl_3_ salts (1.0 and 3.0 equivalents) and 3.0 equivalents of ZnCl_2_.

**Figure 9:**
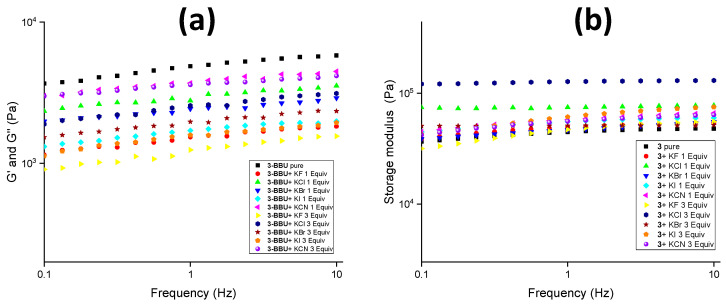
Frequency sweep experiments at 5.0 *wt*/*v*% in DMSO/water (1:1, *v*/*v*) in the presence of various salts of potassium at 25.0 °C with a constant strain of 0.02%, (**a**) **3-BBU**, (**b**) Gelator **3**.

Similarly, we have compared the mechanical strength of **3-BBU** and **3** at 5.0 *wt*/*v*% in DMSO/water (1:1, *v*/*v*) in the presence of salts mentioned above (Table S6). The mechanical strength of the parent gelator **3-BBU** was reduced in the presence of potassium halides and cyanide (Figure 9a), similar to **3-HBU,** and the maximum difference was observed for KF and KI. The presence of these ions enhanced the mechanical strength of gelator **3,** similar to gelator **1** (Figure 9b), and the maximum enhancement was observed for KCl (3.0 equivalents). The experiments were performed with various chloride salts of cations of group IA, group IIA, group III, and transition metals (copper, zinc, and cadmium) revealed that the mechanical strength of **3** increased in the presence of 1.0 and 3.0 equivalents of chlorides salts of sodium, magnesium, calcium, and cadmium (Figure S15a). The gel network collapsed in the presence of ZnCl_2_ (3.0 equivalents), CuCl_2_ (1.0 and 3.0 equivalents), and AlCl_3_ (1.0 and 3.0 equivalents). The experiments performed with parent **3-BBU** in the presence of NaCl, MgCl_2_, and CaCl_2_ (1.0 and 3.0 equivalents) displayed a decrease in mechanical strength (Figure S15b). The gel network of **3-BBU** collapsed in the presence of 1.0 and 3.0 equivalents of ZnCl_2_, CdCl_2_, and AlCl_3_; however, thermo-irreversible metallogels were obtained with CuCl_2_ (1.0 and 3.0 equivalents), with more enhanced mechanical strength than the pure gelator [29].

**Figure 10:**
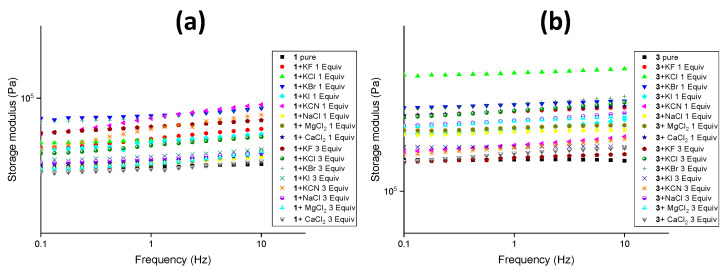
Frequency sweep experiments above MGC in water in the presence of salts at 25.0 °C with a constant strain of 0.02%, (**a**) compound **1** at 2.5 wt%, and (**b**) compound **3** at 3.5 wt%.

The ability of the modified compounds (**1** and **3**) to form hydrogels prompted us to evaluate the stimuli-responsive properties in water in the presence of salts (Table S7), which will enable us to detect anions/cations in water [9,60,61,62]. The rheological experiments on the hydrogels of modified compound **1** (2.5 wt%) and compound **3** (3.5 wt%) in the presence of various salts (Table S7) indicated that potassium halides and cyanide enhanced the mechanical strength of both the hydrogels. A 2-fold increase in mechanical strength was observed for **1** with KCN (1.0 equivalents) (Figure 10a), with KCl for **3** (1.0 equivalents), and with KBr (1.0 equivalents) for both **1** and **3** (Figure 10a,b). Increasing the concentration of the salts (3.0 equivalents) gave similar results but the gel strength was increased compared to the corresponding gelator without additives. We have also performed experiments with sodium halides, nitrates of calcium and magnesium, magnesium sulfate, and chloride salts of calcium, magnesium, strontium, barium, cesium, aluminum, copper, zinc, and cadmium. These results indicated that the presence of these salts enhanced the mechanical strength of compounds **1** and **3**, except for chlorides of aluminum, zinc, cadmium, and copper. Compound **1** (2.5 wt%) formed gel in the presence of 1.0 equivalent of transition metal chlorides and aluminum chloride, but gelation was not observed with 3.0 equivalents of these salts. However, 1.0 equivalent of aluminum chloride did not have a prominent effect on the mechanical strength of gelator **3** (3.5 wt%), but a colloidal solution was observed at 3.0 equivalents. Gels were not obtained for **3** in the presence of 1.0 and 3.0 equivalents of the chloride salts of zinc, cadmium, and copper. These results indicated that the gel network collapsed in the presence of aluminum chloride, cadmium chloride, zinc chloride, and copper chloride in water, but the other salts enhanced the gelation properties (Table S7).

**Figure 11:**
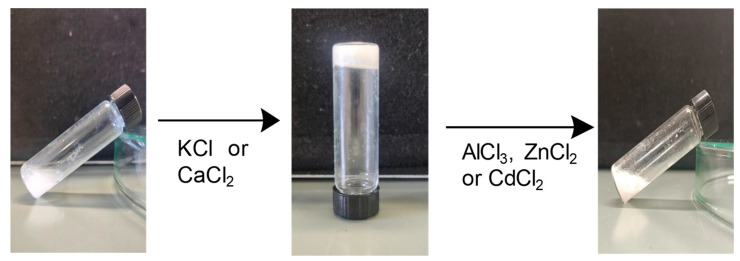
Making and breaking of gels in the presence of salts for compound **1** at 1.0 wt%.

To confirm the role of the salts in gelation properties, we have repeated the experiments at different concentrations, such as minimum gel concentration (MGC) and below MGC. The gelation experiments were performed at MGC with compounds **1** (1.5 wt%) and **3** (2.5 wt%) in the presence of all these salts (Table S7) for rheological studies. The addition of salts (1.0 and 3.0 equivalents), such as potassium halides, potassium cyanide, and chlorides of sodium, magnesium, and calcium, enhanced the mechanical strength of the hydrogels. The mechanical strength was increased (~4-fold) in the presence of the majority of salts for gelator **3** (2.5 wt%), and, for gelator **1** (1.5 wt%), the enhancement of mechanical strength was ~5-fold for the chloride salts of potassium, magnesium, calcium, and potassium fluoride (Figure S16). The experiments repeated with various other salts revealed similar results (enhanced mechanical strength), except for aluminum chloride (1.0 and 3.0 equivalents), and 3.0 equivalents of the transition metal chlorides of cadmium, zinc, and copper ceased gelation. The experiments were performed below MGC to test the ability of these salts to induce gelation in compounds **1** (1.0 wt%) and **3** (2.0 wt%). The results indicated that the salts, such as potassium halides, potassium cyanide, and chlorides of sodium, magnesium, and calcium (1.0 and 3.0 equivalents), induced gelation in **1** and **3** at a concentration below MGC, which was confirmed by vial inversion test, and the gelation properties were analyzed by frequency sweep experiments (Figure S17a,b). However, these gel networks collapsed after adding 3.0 equivalents of chlorides salts of either aluminum, cadmium, zinc, or copper (Figure 11). Furthermore, the presence of AlCl_3_ (1.0 or 3.0 equivalents) and 3.0 equivalents of CdCl_2_, ZnCl_2_, and CuCl_2_ did not induce gelation at concentration below MGC in **1** and **3**.

The enhanced gelation of the *N*-oxide gelators in the presence of salts prompted us to analyze the effect of these salts on compounds **2** and **4**. Compound **2** formed precipitate in water, and the interaction of **4** with the salts were similar to gelators **1** and **3** (Figure S18). We have also performed the experiments with the parent bis(pyridyl urea) compounds in the presence of 1.0 and 3.0 equivalents of these salts, but gelation was not observed due to the poor solubility of the compounds in water. Thus, the structural modification increased the hydrophilic nature in the modified compounds (*N*-oxides), resulting in higher solubility and better interaction of the compounds with ions in water. The interaction of the gelators with the salts favored gelation, which was evident from higher G’ values in the frequency sweep experiment and the gel formation below MGC. Thus, LMWGs acted as stimuli-responsive gelators where gelation was turned ON or OFF in the presence of respective salts/ions.

We further analyzed the morphology of the xerogels of the *N*-oxide gels in water in the presence of potassium fluoride, calcium, and magnesium chlorides using SEM. The xerogel of **1** below MGC (1.0 wt%, partial gel) displayed fibrous morphology with fiber diameter 0.5–2.0 μm (Figure S19a). SEM performed on the xerogels of gelator **1** at 1.0 wt% in the presence of 3.0 equivalents of these salts displayed thin and long dense fibrous bundles (Figure S19b–d). Similarly, we have analyzed the morphology of the xerogels of **3** at 2.0 wt% (below MGC, partial gel) in the presence of the salts (3.0 equivalents). The SEM images displayed plate-shaped morphology in all cases, and no distinct morphological change was identified (Figure S20a–d).

## 3. Materials and Methods

The starting materials and solvents were purchased commercially from Sigma-Aldrich (MEDOR ehf, Reykjavik, Iceland) and TCI-Europe ((Boereveldseweg, Belgium) and were utilized as supplied. Gelation experiments were conducted using deionized water. A Bruker Avance 400 spectrometer (Rheinstetten, Germany) was used to record ^1^H and ^13^C NMR spectra (Figure S21–S32), and the scanning electron microscopy (SEM) (Carl Zeiss, Oberkochen, Germany) was recorded on a Leo Supra 25 microscope. The rheological experiments were performed on Anton Paar modular compact rheometer MCR 302 (Graz, Austria). A Bruker D8 venture (Karlsruhe, Germany) and PANalytical instrument (Almelo, Netherlands) were used to carry out single-crystal X-ray diffraction (SCXRD) and powder X-ray diffraction (XRPD) experiments. Isonicotinoyl azide [63], **3-HBU [29]**, and **3-BBU [48]** were synthesized following the reported procedure, and the analytical data matched with the reported compounds.

### 3.1. Synthesis of Ligands

#### 3.1.1. 1,1′-(Hexane-1,6-diyl)bis(3-(pyridin-4-yl)urea) (**4-HBU**)

A solution of diisocyanatohexane (3.6 g, 21.2 mmol) was dissolved in 30.0 mL of dry acetonitrile and was added dropwise to a stirring solution of 4-aminopyridine (4.0 g, 42.5 mmol) in 50.0 mL of dry acetonitrile. The mixture was heated to reflux for about 48 h, until a thick precipitate was formed. The precipitate was filtered, washed with acetonitrile, and air-dried to obtain the product. Yield: 77.0%. ^1^H NMR (400 MHz, DMSO-*d_6_*) δ (ppm): 11.02 (s, 2H), 8.48 (d, *J* = 6.90 Hz, 4H), 7.80 (d, *J* = 6.64 Hz, 4H), 7.26 (t, *J* = 5.66 Hz, 2H), 3.19–3.09 (m, 4H), 1.50–1.41 (m, 4H), 1.38–1.28 (m, 4H). ^13^C {^1^H} NMR (100 MHz, DMSO-*d_6_*) δ (ppm): 153.77, 153.67, 142.01, 112.10, 39.04, 29.15, 25.93. HRMS (APCI): calcd for C_18_H_25_N_6_O_2_ [M + H]^+^, 357.2034; found, 357.2034.

#### 3.1.2. 1,1′-(Butane-1,4-diyl)bis(3-(pyridin-4-yl)urea) (**4-BBU**)

A solution of isonicotinoyl azide (1.5 g, 10.0 mmol) in toluene (50 mL) was refluxed at 110.0 °C for 2 h under a nitrogen atmosphere and cooled to room temperature. A solution of 1,4-diaminobutane (500 µL, 5.0 mmol) and triethylamine (1.4 mL, 10.0 mmol) in 40 mL toluene was added dropwise to the reaction mixture at room temperature. The resulting pale-yellow solution was further refluxed overnight, and the white precipitate formed was filtered. The residue was washed with methanol and dried in air to obtain the product as a white solid. The compound was recrystallized from methanol/water (3:1, *v*/*v*). Yield: 1.25 g, 76.2%. ^1^H NMR (400 MHz, DMSO-*d_6_*) δ (ppm): 8.87 (s, 2H), 8.27 (dd, *J* = 4.72, 1.64 Hz, 4H), 7.35 (d, *J* = 6.44 Hz, 4H), 6.39 (t, *J* = 5.72 Hz, 2H), 3.11 (m, 4H), 1.45 (m, 4H). ^13^C {^1^H} NMR (100 MHz, DMSO-*d_6_*) δ (ppm): 154.56, 149.65, 147.36, 111.76, 38.78, 27.05. HRMS (APCI): calcd for C_16_H_21_N_6_O_2_ [M + H]^+^, 329.1726; found, 329.1721.

#### 3.1.3. 3,3′-(((Hexane-1,6-diylbis(azanediyl))bis(carbonyl))bis(azanediyl))bis(pyridine 1-oxide)) (**1**)

3–chloroperoxybenzic acid (0.42 g, 2.44 mmol) was added in portions to a solution of **3-HBU** (0.3 g, 0.84 mmol) in 5.0 mL DMF, and the solution was stirred overnight at room temperature [30]. The precipitate formed was filtered and washed with cold water and diethyl ether, and the crude product was recrystallized from hot water. Yield: 72.0%. ^1^H NMR (400 MHz, DMSO-*d_6_*) δ (ppm): 8.82 (s, 2H), 8.56 (s, 2H), 7.79 (d, *J* = 6.2 Hz, 2H), 7.24 (dd, J = 8.53, 6.12 Hz, 2H), 7.20–7.14 (m, 2H), 6.39 (t, *J* = 5.63 Hz, 2H), 3.07 (q, *J* = 6.53 Hz, 4H), 1.49–1.38 (m, 4H), 1.35–1.23 (m, 4H). ^13^C {^1^H} NMR (100 MHz, DMSO-*d_6_*) δ (ppm): 154.52, 139.80, 131.56, 128.55, 125.86, 114.47, 39.11, 29.52, 26.05. HRMS (APCI): calcd for C_18_H_24_N_6_NaO_4_ [M + Na]^+^, 411.1751; found, 411.1748.

#### 3.1.4. 4,4′-(((Hexane-1,6-diylbis(azanediyl))bis(carbonyl))bis(azanediyl))bis(pyridine 1-oxide) (**2**)

Compound **2** was synthesized following a similar procedure to compound **1**. The precipitate obtained from the solution of **4-HBU** (0.6 g, 1.68 mmol) and 3–chloroperoxybenzic acid (0.84 g, 4.88 mmol) in 15.0 mL DMF was filtered. The residue was stirred overnight in 0.05 M HCl to remove unreacted starting materials. The mixture was filtered, washed with water, and dried. The product was recrystallized from hot water. Yield: 62.0%. ^1^H NMR (400 MHz, DMSO-*d_6_*) δ (ppm): 10.08 (s, 2H), 8.33 (d, *J* = 7.47 Hz, 4H), 7.62 (d, *J* = 7.47 Hz, 4H), 6.85 (t, *J* = 5.65 Hz, 2H), 3.10 (q, *J* = 6.50 Hz, 2H), 1.48–1.38 (m, 2H), 1.36–1.26 (m, 2H). ^13^C {^1^H} NMR (100 MHz, DMSO-*d_6_*) δ (ppm): 153.99, 144.87, 139.57, 113.58, 39.08, 29.28, 25.93. HRMS (APCI): calcd for C_18_H_24_N_6_NaO_4_ [M + Na]^+^, 411.1751; found, 411.1752.

#### 3.1.5. 3,3′-(((Butane-1,4-diylbis(azanediyl))bis(carbonyl))bis(azanediyl))bis(pyridine 1-oxide) (**3**)

The reaction procedure was similar to compound **1**. 3–chloroperoxybenzic acid (0.94 g, 5.48 mmol) and **3-BBU** (0.6 g, 1.82 mmol) were added to DMF (10.0 mL). The precipitate was filtered and washed with cold water and ether and was recrystallized from THF/water (1:1, *v*/*v*). Yield: 68.0%. ^1^H NMR (400 MHz, DMSO-*d_6_*) δ (ppm): 8.82 (s, 2H), 8.55 (t, *J* = 1.89 Hz, 2H), 7.79 (dt, *J* = 6.17, 1.40 Hz, 2H), 7.26–7.20 (m, 2H), 7.19–7.15 (m, 2H), 6.41 (t, *J* = 5.70 Hz, 2H), 3.10 (q, *J* = 5.7 Hz, 4H), 1.49–1.40 (m, 4H). ^13^C {^1^H} NMR (100 MHz, DMSO-*d_6_*) δ (ppm): 154.53, 139.76, 131.57, 128.55, 125.84, 114.40, 38.90, 27.05. HRMS (APCI): calcd for C_16_H_20_N_6_NaO_4_ [M + Na]^+^, 383.1444; found, 383.1438.

#### 3.1.6. 4,4′-(((Butane-1,4-diylbis(azanediyl))bis(carbonyl))bis(azanediyl))bis(pyridine 1-oxide) (**4**)

Compound **4** was synthesized following a similar procedure to compound **2**. The compound was recrystallized from water (40.0 mg/mL). Yield: 55.0%. ^1^H NMR (400 MHz, DMSO-*d_6_*) δ (ppm): 9.00 (s, 2H), 7.99 (d, *J* = 7.48 Hz, 4H), 7.40 (d, *J* = 7.52 Hz, 4H), 6.40 (t, *J* = 5.88 Hz, 2H), 3.09 (m, 4H), 1.44 (m, 4H). ^13^C {^1^H} NMR (100 MHz, DMSO-*d_6_*) δ (ppm): 154.92, 150.37, 139.04, 114.61, 39.36, 27.53. HRMS (APCI): calcd for C_16_H_20_N_6_O_4_Na [M + Na]^+^, 383.1444; found, 383.1438.

### 3.2. Gelation Studies

Gelation properties were evaluated with all the compounds in various solvents by weighing 10.0 mg of the compound in a standard 7.0 mL vial (ID = 15.0 mm), into which 1.0 mL of the solvent was added, and the vial was sealed. The mixture was then sonicated and slowly heated to obtain a clear solution and was left undisturbed. An inversion test was conducted to confirm the gelation. Gelation tests were also conducted in the mixed aqueous system, and 10.0 mg of the compound was dissolved in 0.5 mL of the appropriate solvent in a standard 7.0 mL vial, followed by the addition of 0.5 mL distilled water. The mixture was sonicated and heated to obtain a transparent solution. The solution was cooled to room temperature, left undisturbed, and a vial inversion test confirmed gel formation. The experiments were repeated with higher concentrations of the compounds (up to 50.0 mg) to test gelation.

#### 3.2.1. Minimum Gel Concentration (MGC)

The MGC was performed in suitable solvents by weighing various concentration of the compounds in a standard 7.0 mL vial and adding 1.0 mL of solvent/solvent mixture. The mixture was sonicated and heated gradually to dissolve the compounds completely and the solution was kept at room temperature for gel formation. The minimum amount of the compound required to form a stable gel after 24 h was recorded as the MGC.

#### 3.2.2. *T_gel_* Experiments

In a 7.0 mL standard vial, the required amount of gelator and 1.0 mL of solvent was added. The mixture was sonicated and heated to dissolve and left undisturbed for gelation. After 24 h, a ball-drop method was performed to observe the gel to sol transition temperature (*T_gel_*). A spherical glass ball was carefully placed on the top of the gel and immersed in an oil bath. A magnetic stirrer and a thermometer were equipped to monitor the temperature as the oil bath was gradually heated at 10.0 °C per minute. As temperature increases, the glass ball slowly gets immersed into the gels, and the temperature at which the ball touched the bottom of the vial was recorded as *T_gel_*.

### 3.3. Rheology

An Anton Paar Modular Compact Rheometer MCR 302 was used to conduct rheological measurements. A 2.5 cm stainless steel parallel plate geometry configuration was used to measure the mechanical strength. Oscillatory measurements were conducted at a constant temperature of 25.0 °C in all cases. A Peltier temperature control hood was used as a solvent trap, which maintained a temperature of 25.0 °C for frequency and amplitude sweeps. Gels were prepared by dissolving an appropriate amount of the corresponding gelator in 1.0 mL of solvent. The experiments were performed after 24 h by scooping ~1.0 mL portion of gel onto the plate. A constant frequency of 1.0 Hz was maintained during amplitude sweep with log ramp strain (Υ) ranges 0.01–100%. The frequency sweep was then carried out between 0.1 and 10.0 Hz within the linear viscoelasticity domain (0.02% strain). The experiments were also conducted in the presence of different salts in water and DMSO/water (1:1, *v*/*v*), with a similar procedure as mentioned above.

### 3.4. Scanning Electron Microscopy (SEM)

SEM was performed on a Leo Supra 25 microscope which analyzed the surface morphologies of the xerogels. Gels of **3-HBU** (4.0 *wt*/*v*%), **3-BBU** (5.0 *wt*/*v*%), **1** (4.0 *wt*/*v*%), and **3** (5.0 *wt*/*v*%) were prepared in DMSO/water (1:1, *v*/*v*). We have also prepared the hydrogels of **1** at 1.5 wt% (also at a higher concentration, 3.0 wt%) and at 2.5 wt% for compound **3**. The gels were filtered after 24 h and dried under a fume hood to obtain the xerogel. A small portion of the xerogel was placed on a pin mount with the carbon tab on top, coated with gold for 5–6 min to prevent charging of the surface, and was loaded, and the images were recorded at an operating voltage of 3.0 kV with a working distance 3–4 mm. An in-lens detector was used to record the SEM images. SEM of the xerogel of gelator **1** and **3** in the presence of certain salts of potassium, magnesium, and calcium in water was also recorded.

### 3.5. Single Crystal X-ray Diffraction

Single crystal of compound **1** was obtained by the slow evaporation of 10.0 mg of the compound in 2.0 mL of DMSO/water (1:1 *v*/*v*) to obtain needle-shaped crystals. Similarly, for compound **3**, a solvent composition of 2.0 mL of DMSO/water (1:2 *v*/*v*) of 10.0 mg of the compound was utilized to obtain the single crystals with needle-shaped morphology within a span of 24 h. X-ray quality crystals of **4-BBU** were obtained from a methanol/water (3:1, *v*/*v*) solution (10.0 mg/mL), and the slow evaporation of 40.0 mg of the compound **4** in 1.0 mL of water yielded needle-shaped single crystals. A Bruker D8 Venture (Photon100 CMOS detector) diffractometer equipped with Cryostream (Oxford Cryosystems) open-flow nitrogen cryostats was used for the X-ray analysis. The data was collected using MoKα radiation (λ = 0.71073 Å) for the crystals **1** at 293(2) K, **4-BBU** and **4** at 296(2) K, and compound **3** at 150(2) K. The unit cell determination, data collection, data reduction, structure solution/refinement, and empirical absorption correction (SADABS) were carried out using Apex III software (Bruker AXS: Madison, WI, USA, 2015). The structure was solved by direct method and refined by the full-matrix least-squares on F^2^ for all data using SHELXTL version 2017/1 (University of Göttingen, Göttingen, Germany) [64]. All non-disordered nonhydrogen atoms were refined anisotropically, and the hydrogen atoms were placed in the calculated positions and refined using a riding model, except for the solvent water molecules in structures **1**, **3**, and **4**, where the hydrogen atoms were located on the Fourier map and refined. The crystallographic data and hydrogen bonding parameters are given in Tables S3 and S4 (see Supplementary Materials). The crystallographic data was deposited at the Cambridge Crystallographic Data Center, which can be obtained free of charge, and the CCDC numbers are 2113251–2113254.

### 3.6. X-ray Powder Diffraction

The bulk crystals of compounds **1** and **3** were obtained by the slow evaporation of the solution of **1** and **3** (20.0 mg in 5.0 mL DMSO/water 1:1, *v*/*v*). The crystals were filtered, dried in the air, and ground to a fine powder. The xerogels compound **1** were prepared from corresponding gels made in water at 2.0 wt% and DMSO/water (1:1, *v*/*v*) at 4.5 *wt*/*v*%. Gels were further filtered and dried in a fume hood to obtain the xerogel. A similar strategy was employed to prepare xerogels of compound **3** in water at 2.5 wt% and DMSO/water (1:1, *v*/*v*) at 5.0 *wt*/*v* %. XRPD studies with **3-BBU** were performed with bulk crystals obtained from THF/water (1:1, *v*/*v*) and xerogels at 5.0 *wt*/*v*% in EG/water (3:7, *v*/*v*) and DMSO/water (1:1, *v*/*v*) to compare with the reported crystal structure. XRPD was also performed with as-synthesized compound **4** and **4-BBU** and was matched with the simulated data. All experiments were carried out in a PANalytical instrument with Cu anode (Almelo, Netherlands), between 2θ from 4.0 to 50.0, and a step size of 0.02.

## 4. Conclusions

The functional group modification of bis(pyridyl urea) compounds to corresponding bis(pyridyl-*N*-oxide urea) was performed to tune the gelation properties of LMWGs by altering the specific non-bonding interaction. The analysis of the gelation properties revealed that the modification of the functional groups induced hydrogelation in 3-pyridyl-based bis(urea *N*-oxides) compounds (**1** and **3**). The structural modification enhanced the mechanical stabilities of the modified compounds (**1** and **3**), but the thermal stability of the gels in DMSO/water (1:1, *v*/*v*) and EG/water (3:7, *v*/*v*) were lower than the parent gelators. The morphologies of the gels were analyzed by SEM, which indicated that plate-shaped morphologies were observed in most cases, except for the hydrogel **1** with fibrous morphology. Single crystal X-ray diffraction revealed that the structural modification resulted in the alteration of non-bonding interactions and the modified compounds displayed N—H⋯O interactions involving the urea and pyridyl-*N*-oxide moieties, similar to complementary urea hydrogen bonding. The stimuli-responsive properties of the parent and modified gelators were studied in the presence of various salts. The addition of salts resulted in enhanced mechanical strength of the modified *N*-oxide gels, but a reverse behavior was observed for the parent gelators. The anion/cation sensing properties of the gelators in water were evaluated at different gelator concentrations, and the results indicated that the majority of the salts enhanced the gelation properties, but the gel network collapsed in the presence of aluminum chloride, cadmium chloride, zinc chloride, and copper chloride. Gelation was induced by various salts/ions in the modified compounds at concentration below MGC, with enhanced mechanical strength. Thus, these stimuli responsive LMWGs can act as sensors, and the gelation can be turned ON/OFF in the presence of respective salts/ions.

## Figures and Tables

**Table 1:** Determination of sol-gel transition temperature (*T_gel_*).

Solvent	Tgel (°C)			
	3-HBU	3-BBU	1	3
Water	---	---	85.8 ^*^	67.8 ^*^
Ethylene glycol	69.9 ^*^	---	---	---
DMSO: water (1:1, *v*/*v*)	94.4 ^#^	---	62.8 ^#^	---
	99.3 ^##^	96.6 ^##^	65.9 ^##^	61.9 ^##^
DMF: water (1:1, *v*/*v*)	84.8 ^##^	93.3 ^##^	---	55.6 ^##^
EG: water (3:7, *v*/*v*)	93.9 ^#^	---	59.1 ^#^	---
	98.4 ^##^	104.8 ^##^	64.2 ^##^	68.7 ^##^

^*^ = 3.0 *wt*/*v*%, ^#^ = 4.0 *wt*/*v*%, ^##^ = 5.0 *wt*/*v*%.

## Data Availability

Not applicable.

## Supplementary Materials

The following are available online. Scheme S1: Synthetic route for the parent gelators **3-HBU** and **4-HBU**; Scheme S2: Synthetic route for the parent gelators **3-BBU** and **4-BBU**; Scheme S3: Synthetic route for the *N*-oxides; Table S1: Gelation details; Table S2: Determination of Minimum Gel Concentration (MGC); Table S3: Crystal data; Table S4: Hydrogen bonding parameters; Table S5: *T_gel_* experiments in the presence of salts in DMSO/water (1:1, *v*/*v*); Table S6: Stimuli-responsive properties of the parent and modified compounds: comparing the G’ values in DMSO/water (1:1, *v*/*v*)*; Table S7: Stimuli-responsive properties of the modified compounds **1**, **3**, and **4**: comparing the G’ values in water*; Figure S1: Amplitude sweep experiments with gels of **3-HBU** and **1** (5.0 *wt*/*v*%) at 25.0 °C with a constant frequency of 1.0 Hz; Figure S2: Amplitude sweep experiments with gels of **3-BBU** and **3** (5.0 *wt*/*v*%) at 25.0 °C with a constant frequency of 1.0 Hz; Figure S3: Frequency sweep experiments with gels of **3-HBU** and **1** (5.0 *wt*/*v*%) at 25.0 °C with a constant strain of 0.02%; Figure S4: SEM images of xerogels in DMSO/water (1:1, *v*/*v*) at 5.0 *wt*/*v*% (a) **3-BBU**, and (b) **3**; Figure S5: (a) One-dimensional chains interconnected by O—H⋯O interactions between the pyridyl-N-oxide moiety and the solvent water molecule in gelator **1** and (b) two-dimensional porous architecture of gelator **3** with entrapped water molecules; Figure S6: (a) Molecular structure of **4-BBU** and (b) one-dimensional chain of **4-BBU** formed by N⋯H—O interactions; Figure S7: (a) Molecular structure of **4**·2H_2_O, (b) bifurcated hydrogen bonding of the urea and pyridyl-*N*-oxide moieties resulting in two-dimensional hydrogen-bonded sheet, and (c) solvent water molecules entrapped in the cavities of the two-dimensional sheets; Figure S8: Comparison of the simulated pattern from single-crystal X-ray structure of compound **1** with the XRPD pattern of the bulk crystals obtained from DMSO/water, xerogel from DMSO/water (1:1, *v*/*v*, 4.0 *wt*/*v*%), and xerogel from water at 3.0 wt%; Figure S9: Comparison of the simulated pattern from single-crystal X-ray structure of compound **3** with the XRPD pattern of the xerogel from water at 3.0 wt%; Figure S10: Comparison of the simulated pattern from single-crystal X-ray structure of compound **3-BBU** with the XRPD pattern of as-synthesized bulk crystals obtained from THF/water, xerogel from DMSO/water (1:1, *v*/*v*); Figure S11: Comparison of the simulated pattern from single-crystal X-ray structure of **4-BBU** with the XRPD pattern of the bulk crystals obtained from MeOH/water; Figure S12: Comparison of the simulated pattern from single-crystal X-ray structure of **4**: with the XRPD pattern of bulk crystals obtained from water; Figure S13: Frequency sweep experiments at 4.0 *wt*/*v*% in DMSO/water (1:1, *v*/*v*) in the presence of various salts of chlorides at 25.0 °C with a constant strain of 0.02%, (a) gelator **1**, and (b) **3-HBU**; Figure S14: Gel network of **1** collapsing in the presence of 3.0 equivalents of ZnCl_2_ or AlCl_3_; Figure S15: Frequency sweep experiments at 5.0 *wt*/*v*% in DMSO/water (1:1, *v*/*v*) in the presence of various salts of chlorides at 25.0 °C with a constant strain of 0.02%, (a) gelator **3**, and (b) **3-BBU**; Figure S16: Frequency sweep experiments at MGC in water in presence of salts at 25.0 °C with a constant strain of 0.02%, (a) compound **1** at 1.5 wt%, and (b) compound **3** at 2.5 wt%; Figure S17: Frequency sweep experiments below MGC in water in presence of salts at 25.0 °C with a constant strain of 0.02%, (a) compound **1** at 1.0 wt%, and (b) compound **3** at 2.0 wt%; Figure S18: Frequency sweep experiments with compound **4** in the presence of 3.0 equivalents of the salts in water at 25.0 °C with a constant strain of 0.02%, (a) 7.0 wt%, and (b) 5.0 wt%; Figure S19: SEM images of xerogels of **1** obtained from pure water at 1.0 wt% (below MGC), (a) partial gel, and in the presence of 3.0 equivalents of (b) potassium fluoride, (c) magnesium chloride, and (d) calcium chloride; Figure S20: SEM images of xerogels of **3** obtained from pure water at 2.0 wt% (below MGC), (a) partial gel, and in the presence of 3.0 equivalents of (b) potassium fluoride, (c) magnesium chloride, and (d) calcium chloride; Figure S21: ^1^H NMR spectrum of compound **4-HBU**; Figure S22: ^13^C NMR spectrum of compound **4-HBU**; Figure S23: ^1^H NMR spectrum of compound **4-BBU**; Figure S24: ^13^C NMR spectrum of compound **4-BBU**; Figure S25: ^1^H NMR spectrum of compound **1;** Figure S26: ^13^C NMR spectrum of compound **1**; Figure S27: ^1^H NMR spectrum of compound **2**; Figure S28: ^13^C NMR spectrum of compound **2**; Figure S29: ^1^H NMR spectrum of compound **3**; Figure S30: ^13^C NMR spectrum of compound **3**; Figure S31: ^1^H NMR spectrum of compound **4**; Figure S32: ^13^C NMR spectrum of compound **4**.

## Sample Availability

Samples of the compounds are available in limited quantity from the authors.
