# Supplementary material for: Making and Breaking of Gels: Stimuli-Responsive Properties of Bis(Pyridyl-N-oxide Urea) Gelators
Source: Molecules. 2021 Oct 24;26(21):6420. doi: 10.3390/molecules26216420 (PMC8587056; doi:10.3390/molecules26216420)
Supplement: Supplementary file 1 [file molecules-26-06420-s001.zip › molecules-1425212-supplementary.pdf]

Supporting Information

Making and Breaking of Gels: Stimuli-responsive Properties of Bis(Pyridyl-*N*-oxide Urea) Gelators

Sreejith Sudhakaran Jayabhavan, Dipankar Ghosh and Krishna K. Damodaran*

**1.**	Synthetic scheme	2
**2.**	Gelation studies	3
**3.**	Rheology	4
**4.**	Scanning electron microscopy	6
**5.**	X-ray crystallography	7
**6.**	X-ray powder diffraction	12
**7.**	Physical properties in the presence of salts...	15
**8.**	NMR Spectra	26

1. Synthetic scheme

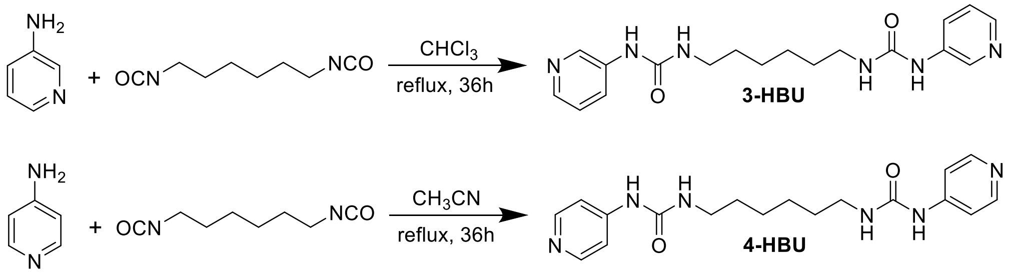

Scheme S1. Synthetic route for the parent gelators **3-HBU** and **4-HBU**.

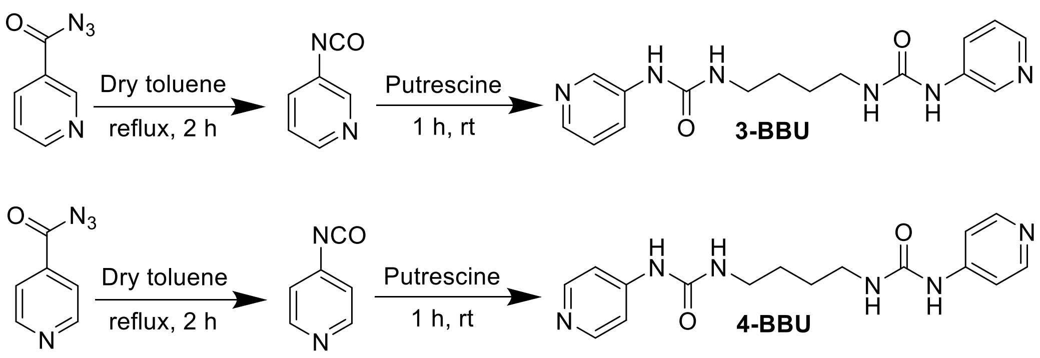

Scheme S2. Synthetic route for the parent gelators **3-BBU** and **4-BBU**.

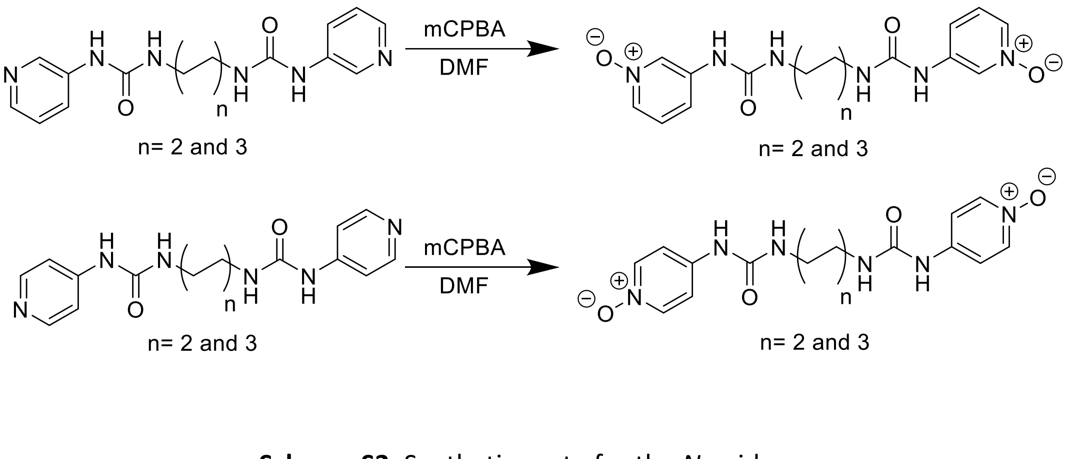

Scheme S3. Synthetic route for the *N*-oxides.

2. Gelation studies

The gelation studies were performed by taking a 10.0 mg of the compounds in 1.0 mL of the solvent/solvent mixture. The mixture was heated and sonicated and cooled to room temperature. When a clear solution was observed, another 10.0 mg of the compounds was added and the procedure was repeated until a gel was obtained, or up to 50.0 mg/mL concentration. The observations were recorded 24 h after the experiments.

Table S1: Gelation details

**Solvents**	**3-HBU**	**4-HBU**	**3-BBU**	**4-BBU**	**1**	**2**	**3**	**4**
water	I	I	I	I	G*	Ppt	G**	PG ##
DMSO	S	S	S	S	S	Ppt	S	I
DMF	S	S	S	S	S	Ppt	S	I
Nitrobenzene	S	S	S	S	I	I	Ppt	I
1,2-dibromoethane	I	I	I	I	I	I	I	I
THF	I	I	I	I	I	I	I	I
Acetonitrile	I	I	I	I	I	I	I	I
o-xylene	I	I	I	I	I	I	I	I
m-xylene	I	I	I	I	I	I	I	I
p-xylene	I	I	I	I	I	I	I	I
Mesitylene	I	I	I	I	I	I	I	I
Ethanol	Ppt	Ppt	Ppt	Ppt	I	I	I	I
1-propanol	Ppt	Ppt	Ppt	Ppt	I	I	I	I
1-butanol	Ppt	Ppt	Ppt	Ppt	I	I	I	I
1-pentanol	Ppt	Ppt	Ppt	Ppt	I	I	I	I
Benzyl alcohol	S	S	S	S	S	S	S	I
Ethylene glycol	G**	S	Cry	S	Ppt	S	Ppt	Ppt
DMSO:water (1:1, v/v)	G#	C	G##	C	G#	Ppt	G##	Ppt
DMF:water (1:1, v/v)	G##	C	G##	C	Ppt	Ppt	G##	Ppt
EG:water (3:7, v/v)	G#	C	G##	C	G#	Ppt	G##	S

G= gel, I= insoluble, S= solution, C= colloid, Ppt= precipitate, PG= partial gel, Cry= crystals, *=2.0 wt/v%, **= 3.0 wt/v%, #= 4.0 wt/v%, ##= 5.0 wt/v%.

Table S2: Determination of Minimum Gel Concentration (MGC)

**Solvent**	**MGC (wt/v%)**			
	**3-HBU**	**3-BBU**	**1**	**3**
water	---	---	1.6	2.5
Ethylene glycol	2.8	---	---	---
DMSO:water (1:1, v/v)	4.0	4.8	3.8	4.5
DMF:water (1:1, v/v)	4.5	4.8	---	4.5
EG:water (3:7, v/v)	3.8	4.5	3.8	4.0

3. Rheology

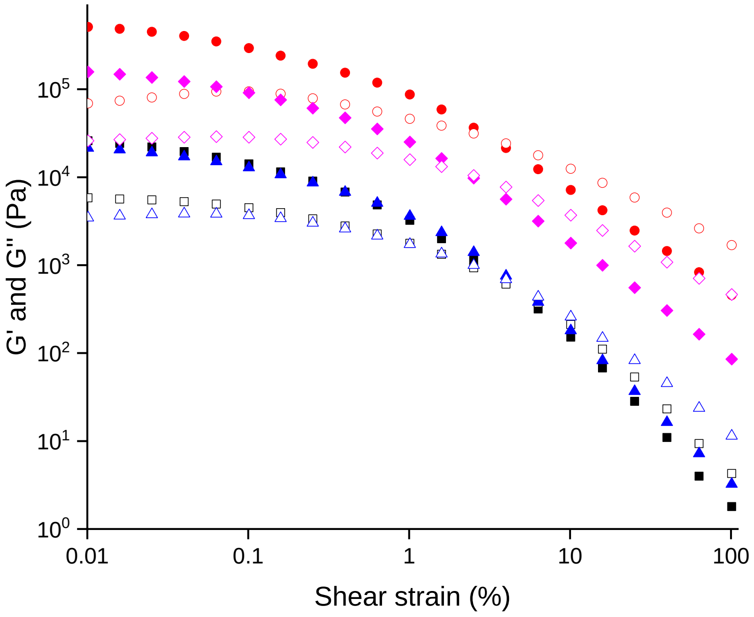

Figure S1. Amplitude sweep experiments with gels of **3-HBU** and **1** (5.0 wt/v%) at 25.0 °C with a constant frequency of 1.0 Hz. Color codes: In EG/water (3:7, v/v) **3-HBU** G′ (■) and G′′ (□), compound **1** G′ (●) and G′′ (○), and in DMSO/water (1:1, v/v) **3-HBU** G′ (▲) and G′′ (Δ), and compound **1** G′ (♦) and G′′ (◊).

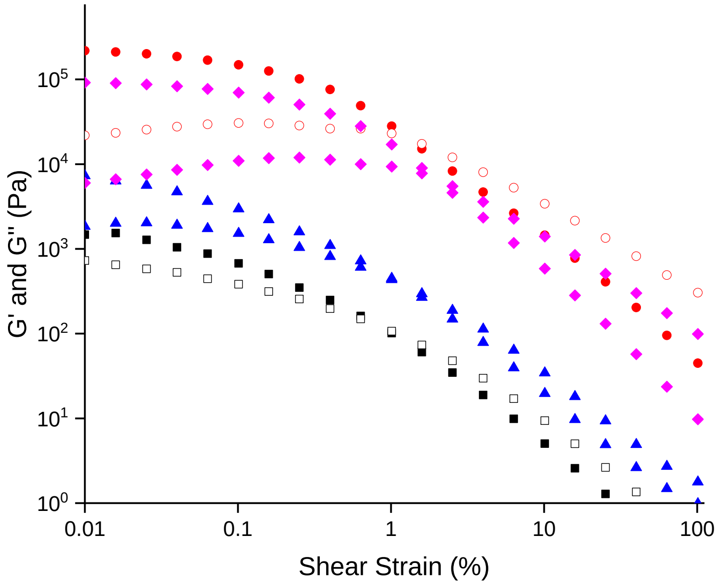

Figure S2. Amplitude sweep experiments with gels of **3-BBU** and **3** (5.0 wt/v%) at 25.0 °C with a constant frequency of 1.0 Hz. Color codes: In EG/water (3:7, v/v) **3-BBU** G′ (■) and G′′ (□), compound **3** G′ (●) and G′′ (○), and in DMSO/water (1:1, v/v) **3-BBU** G′ (▲) and G′′ (Δ), and compound **3** G′ (♦) and G′′ (◊).

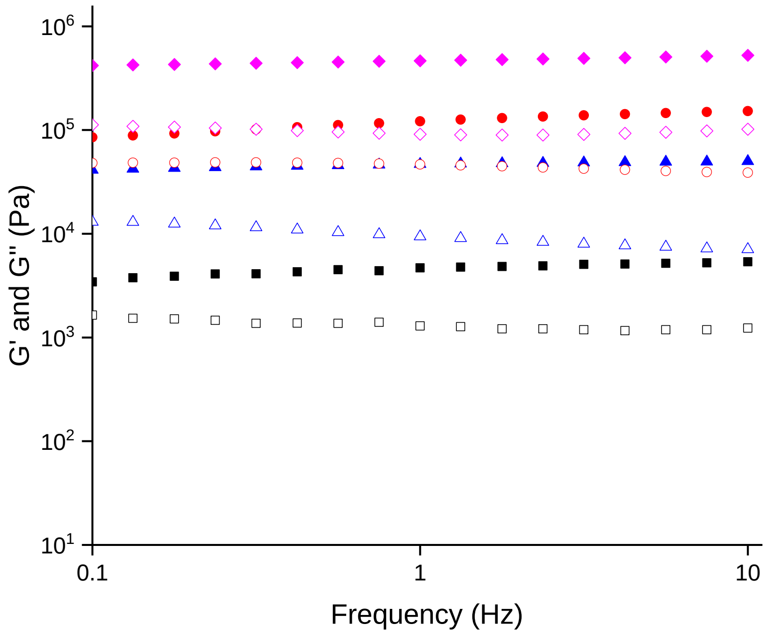

Figure S3. Frequency sweep experiments with gels of **3-HBU** and **1** (5.0 wt/v%) at 25.0 °C with a constant strain of 0.02%. Color codes: In EG/water (3:7, v/v) **3-HBU** G′ (■) and G′′ (□), compound **1** G′ (●) and G′′ (○), and in DMSO/water (1:1, v/v) **3-HBU** G′ (▲) and G′′ (Δ), and compound **1** G′ (♦) and G′′ (◊).

4. Scanning electron microscopy

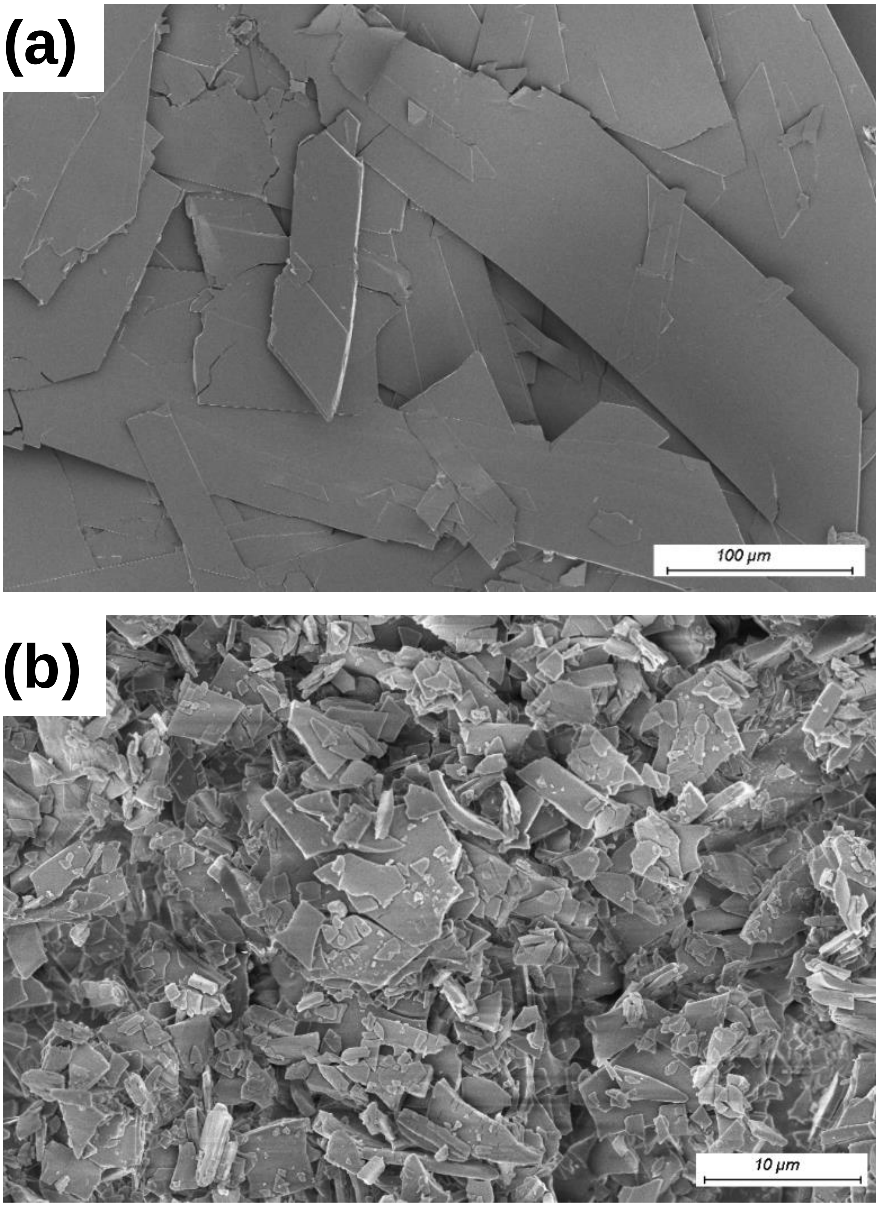

Figure S4. SEM images of xerogels in DMSO/water (1:1, v/v) at 5.0 wt/v%, (a) **3-BBU**, and (b) **3**.

5. X-ray crystallography

Table S3: Crystal data

**Crystal data**	**1.H_2O**	**3.2H_2O**	**4-BBU**	**4.2H_2O**
Empirical formula	$C_{18}H_{26}N_6O_5$	$C_{16}H_{24}N_6O_6$	$C_{16}H_{20}N_6O_2$	$C_{16}H_{24}N_6O_6$
Color	Colorless	Colorless	Colorless	Colorless
Formula weight	406.45	396.41	328.38	396.41
Crystal size (mm)	0.40 x 0.16 x 0.12	0.16 x 0.09 x 0.06	0.50 x 0.01 x 0.08	0.44 x 0.072 x 0.065
Crystal system	Monoclinic	Monoclinic	Monoclinic	Monoclinic
Space group	C2/c	$P2_1/n$	$P2_1/n$	$P2_1/n$
a (Å)	13.860(3)	4.9830(4)	4.9644(3)	4.7570(3)
b (Å)	5.281(2)	10.8892(9)	15.5022(13)	11.5219(7)
c (Å)	25.954(5)	16.1948(14)	10.5214(9)	16.9900(10)
α (°)	90	90	90	90
β (°)	94.00(3)	90.699(3)	96.893(3)	93.104(2)
γ (°)	90	90	90	90
Volume (Å^3)	1895.1(9)	878.68(13)	803.87(11)	929.85(10)
Z	4	2	2	2
$D_{calc.}$(g/cm^3)	1.425	1.498	1.357	1.416
F(000)	864	420	348	420
μ (mm^{-1}) MoK_α	0.106	0.116	0.094	0.110
Temperature (K)	293(2)	150(2)	296(2)	296(2)
Reflections collected/ unique/observed [I>2σ(I)]	22066/2564/ 2207	16106/2010/ 1574	18333/1713/ 1324	33014/1845/ 1357
Data/restraints/parameters	2564/0/136	2010/0/135	1713/0/109	1845/0/135
Goodness of fit on F^2	1.036	1.049	1.096	1.071
Final R indices [I>2σ(I)]	R_1 = 0.0395 wR_2 = 0.0985	R_1 = 0.0406 wR_2 = 0.0891	R_1 = 0.0434 wR_2 = 0.0993	R_1 = 0.0410 wR_2 = 0.0858
R indices (all data)	R_1 = 0.0525 wR_2 = 0.1033	R_1 = 0.0602 wR_2 = 0.0970	R_1 = 0.0636 wR_2 = 0.1094	R_1 = 0.0677 wR_2 = 0.0951

Table S4: Hydrogen bonding parameters

Compound 1.H_2O						
No.	Donor—H···Acceptor	D–H(Å)	H···A(Å)	D···A(Å)	∠D—H···A(°)	Symmetry operation
1	O(15)—H(1)···O(1)	0.858(18)	1.983(18)	2.8224(14)	165.8(16)	-1/2+x,1/2+y,z
2	N(8)—H(8)···O(1)	0.86	2.16	2.8749(16)	140	-1/2+x,1/2+y,z
3	N(11)—H(11)···O(1)	0.86	2.25	2.9531(17)	139	-1/2+x,1/2+y,z
4	C(3)—H(3)···O(15)	0.93	2.42	3.0953(17)	130	1/2+x,1/2+y,z
Compound 3.2H_2O						
No.	Donor—H···Acceptor	D–H (Å)	H···A (Å)	D···A (Å)	∠D—H···A (°)	Symmetry operation
1	N(8)—H(8)···O(14)	0.88	1.95	2.8011(17)	163	x,y,z
2	N(11)—H(11)···O(1)	0.88	2.21	3.0246(16)	153	-1/2+x,1/2-y,1/2+z
3	O(14)—H(14A)···O(1)	0.88(2)	2.05(2)	2.9013(18)	162(2)	3/2-x,1/2+y,1/2-z
4	O(14)—H(14B)···O(1)	0.94(3)	1.90(3)	2.8329(18)	171(2)	1/2-x,1/2+y,1/2-z
5	C(4)—H(4)···O(10)	0.95	2.41	3.3514(18)	174	3/2-x,1/2+y,1/2-z
Compound 4-BBU						
No.	Donor—H···Acceptor	D–H (Å)	H···A (Å)	D···A (Å)	∠D—H···A (°)	Symmetry operation
1	N(7)—H(7)···N(1)	0.86	2.27	2.986(2)	141	-1/2+x,3/2-y,1/2+z
2	N(10)—H(10)···O(9)	0.86	2.17	2.9871(15)	157	-1+x,y,z
Compound 4.2H_2O						
No.	Donor—H···Acceptor	D–H (Å)	H···A (Å)	D···A (Å)	∠D—H···A (°)	Symmetry operation
1	N(8)—H(8)···O(1)	0.86	1.96	2.7887(18)	163	3/2-x,-1/2+y,1/2-z
2	N(11)—H(11)···O(1)	0.86	2.24	2.9988(18)	147	3/2-x,-1/2+y,1/2-z
3	O(14)—H(14A)···O(1)	1.10(3)	1.69(3)	2.788(2)	176.4(19)	2-x,1-y,1-z
4	O(14)—H(14B)···O(10)	0.89(3)	2.01(3)	2.889(2)	169(3)	x,y,z
5	C(6)—H(6)···O(14)	0.93	2.58	3.201(2)	125	1/2+x,1/2-y,-1/2+z

(a)

(b)

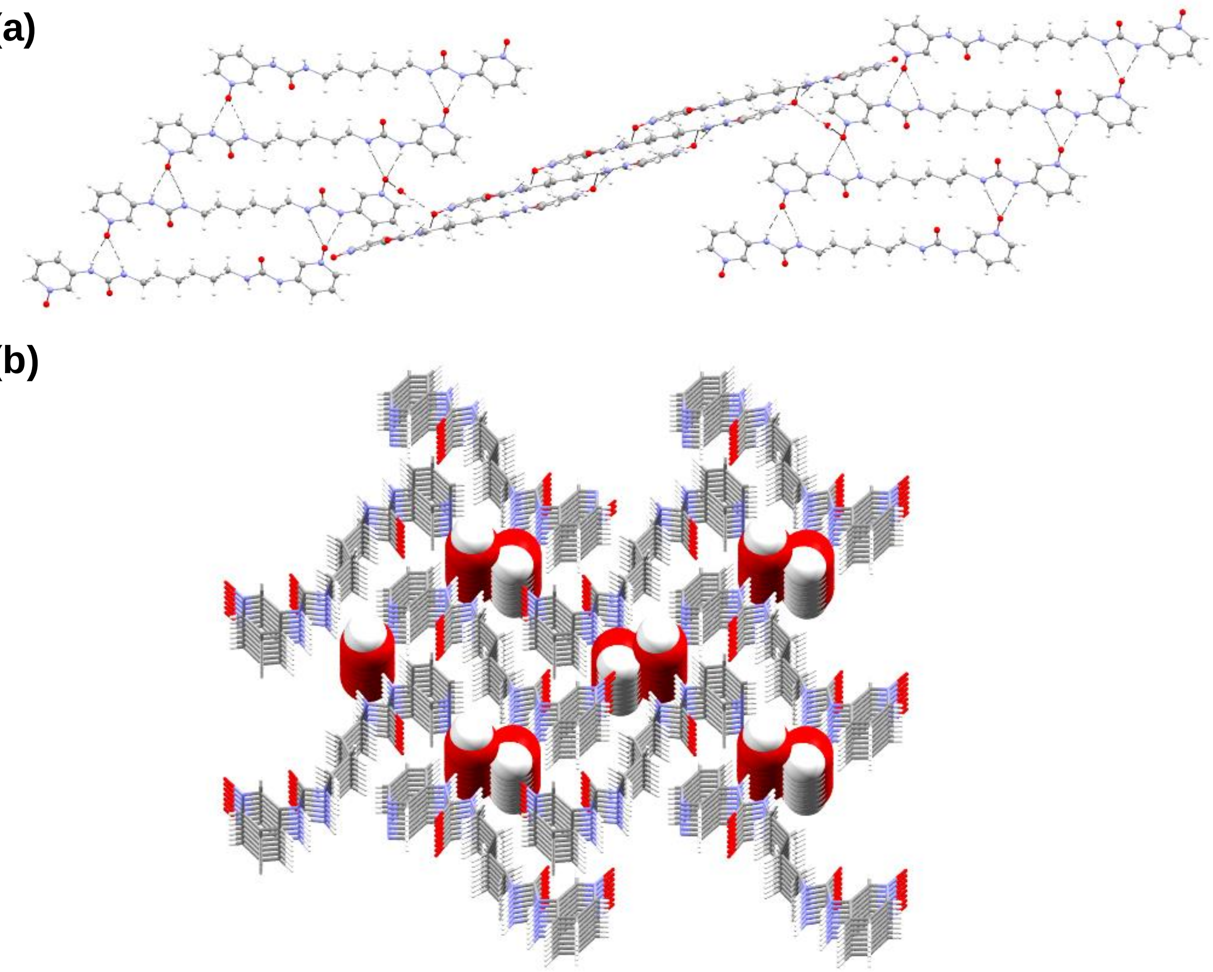

Figure S5. (a) One-dimensional chains interconnected by O—H···O interactions between the pyridyl-N-oxide moiety and the solvent water molecule in gelator **1** and (b) two-dimensional porous architecture in gelator **3** with entrapped water molecules.

(a)

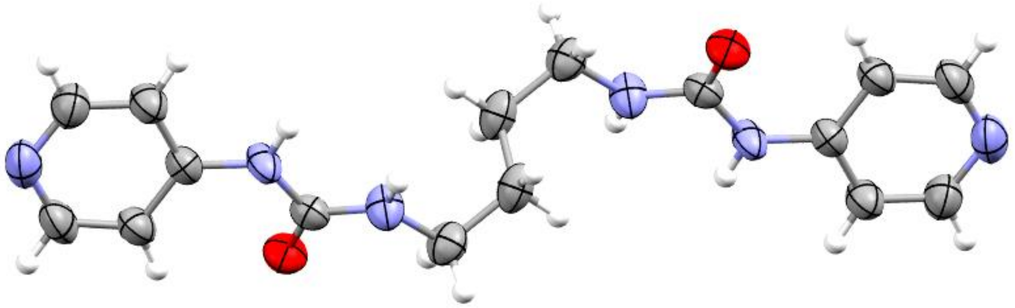

(b)

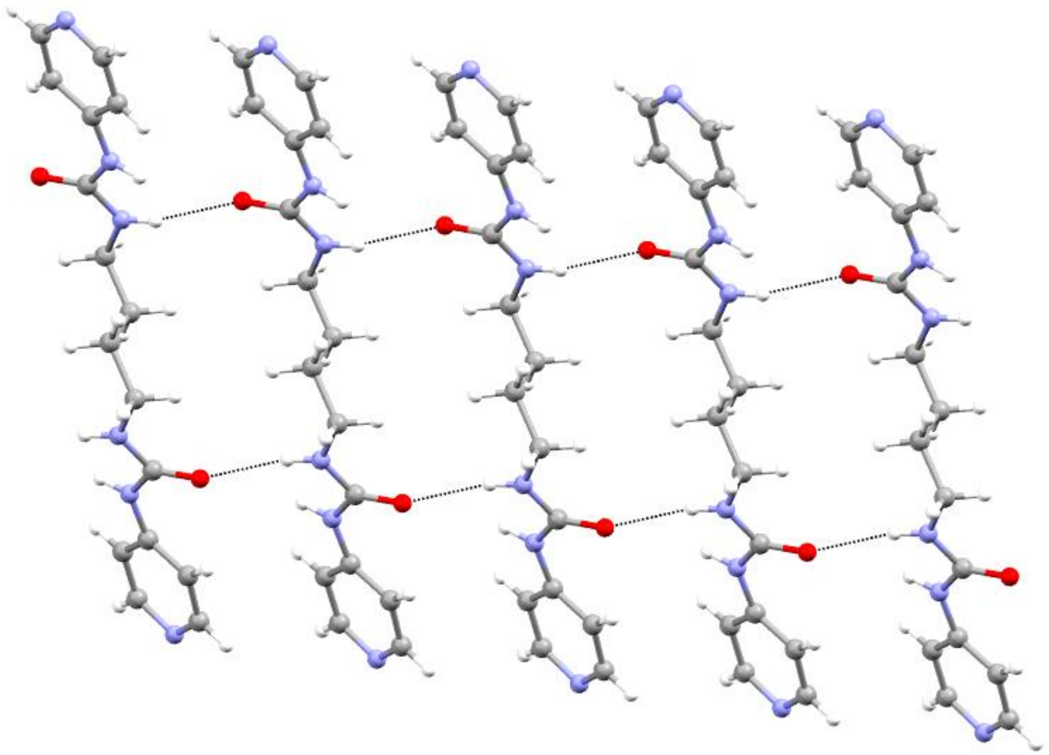

Figure S6. (a) Molecular structure of **4-BBU** and (b) one-dimensional chain of **4-BBU** formed by N···H—O interactions.

(a)

(b)

(c)

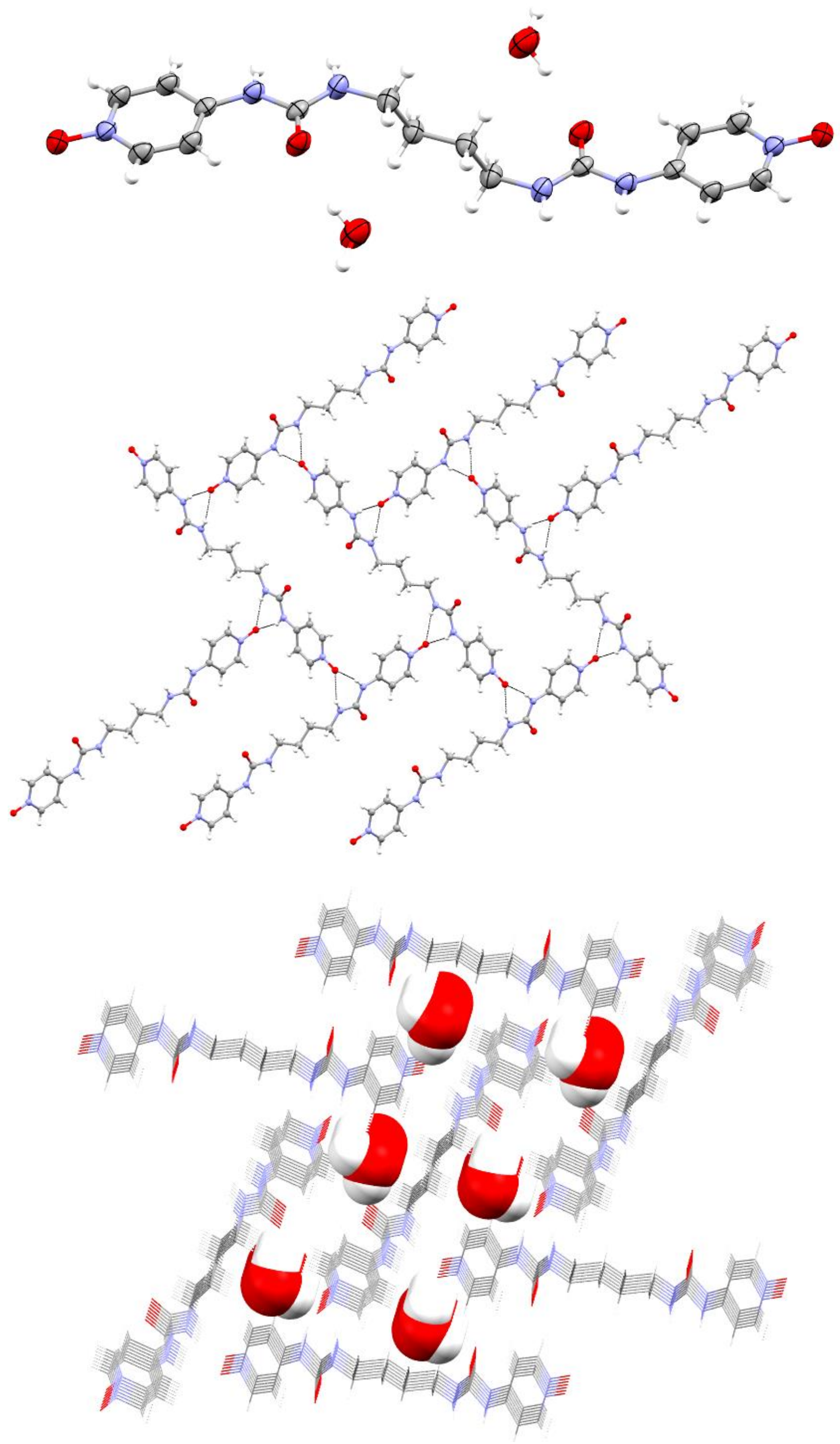

Figure S7. (a) Molecular structure of **4**•$2H_2O$, (b) bifurcated hydrogen bonding of the urea and pyridyl-*N*-oxide moieties resulting in two-dimensional hydrogen-bonded sheet and (c) solvent water molecules entrapped in the cavities of the two-dimensional sheets.

6. X-ray powder diffraction

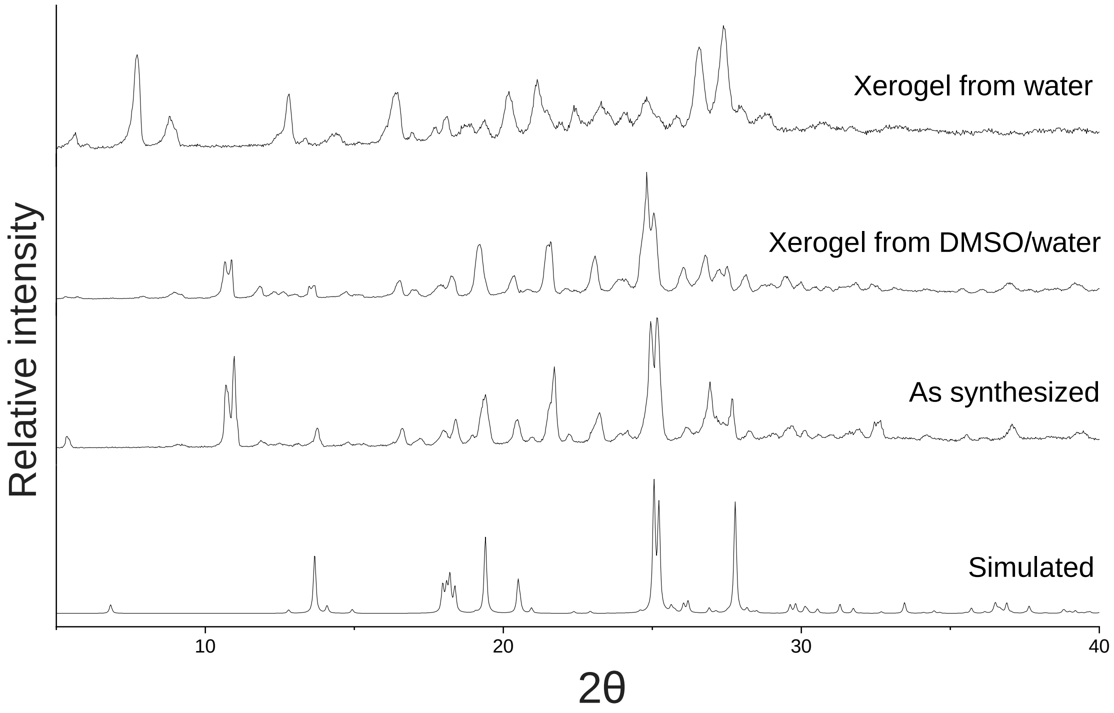

Figure S8. Comparison of the simulated pattern from single-crystal X-ray structure of compound **1** with the XRPD pattern of the bulk crystals obtained from DMSO/water, xerogel from DMSO/water (1:1, v/v, 4.0 wt/v%), and xerogel from water at 3.0 wt%.

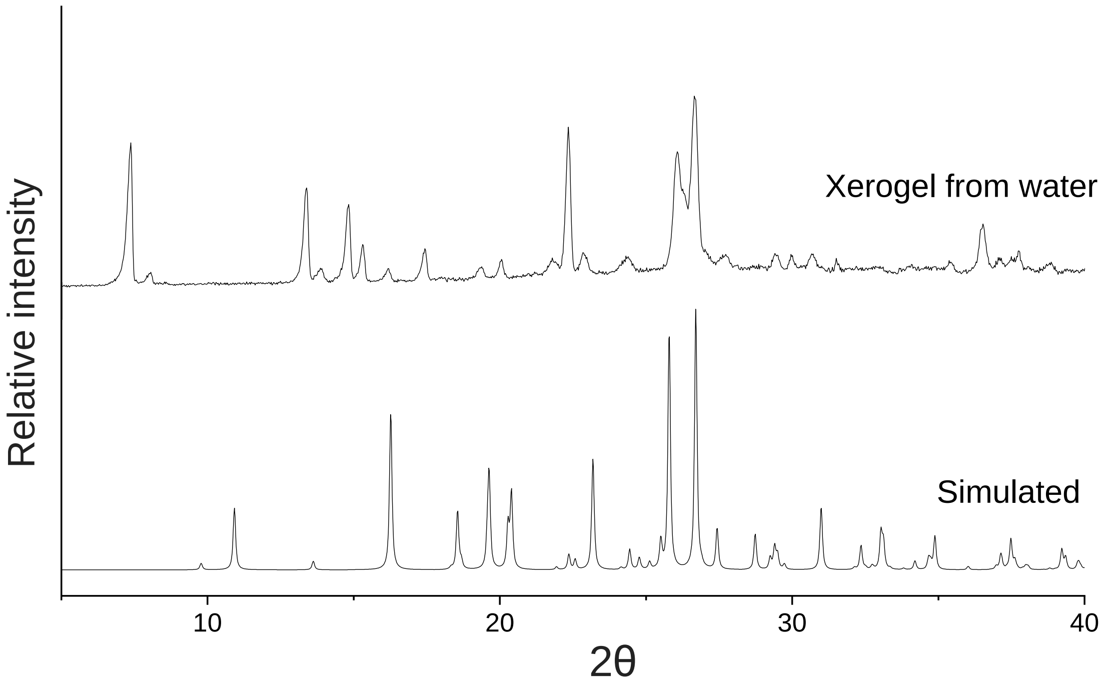

Figure S9. Comparison of the simulated pattern from single-crystal X-ray structure of compound **3** with the XRPD pattern of the xerogel from water at 3.0 wt%.

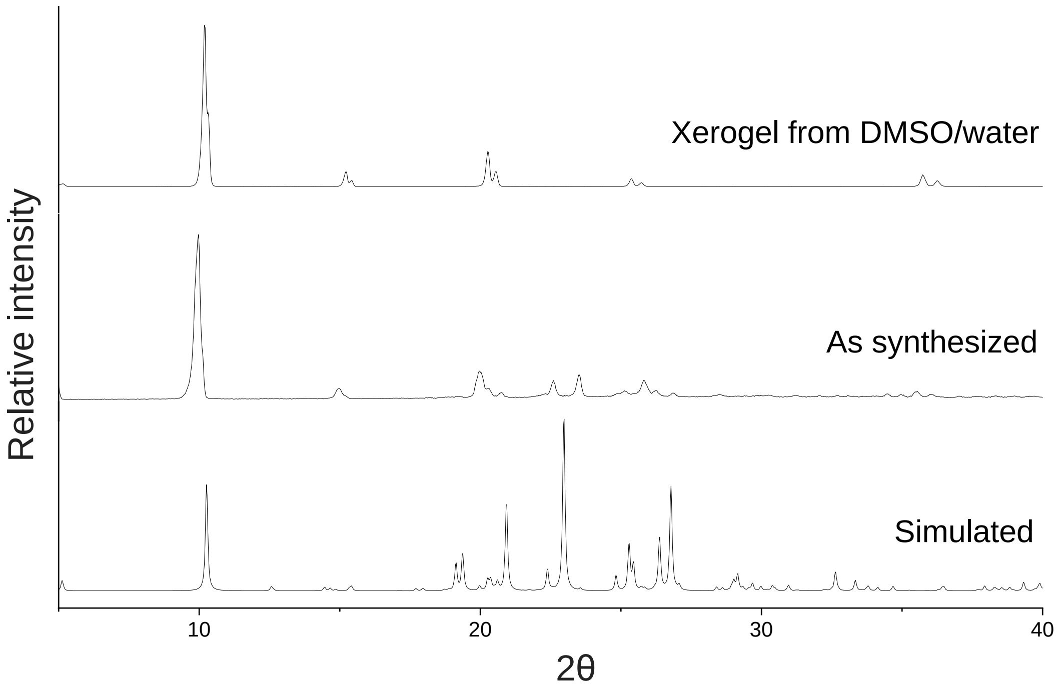

Figure S10. Comparison of the simulated pattern from single-crystal X-ray structure of compound **3-BBU** with the XRPD pattern of as-synthesized bulk crystals obtained from THF/water, xerogel from DMSO/water (1:1, v/v).

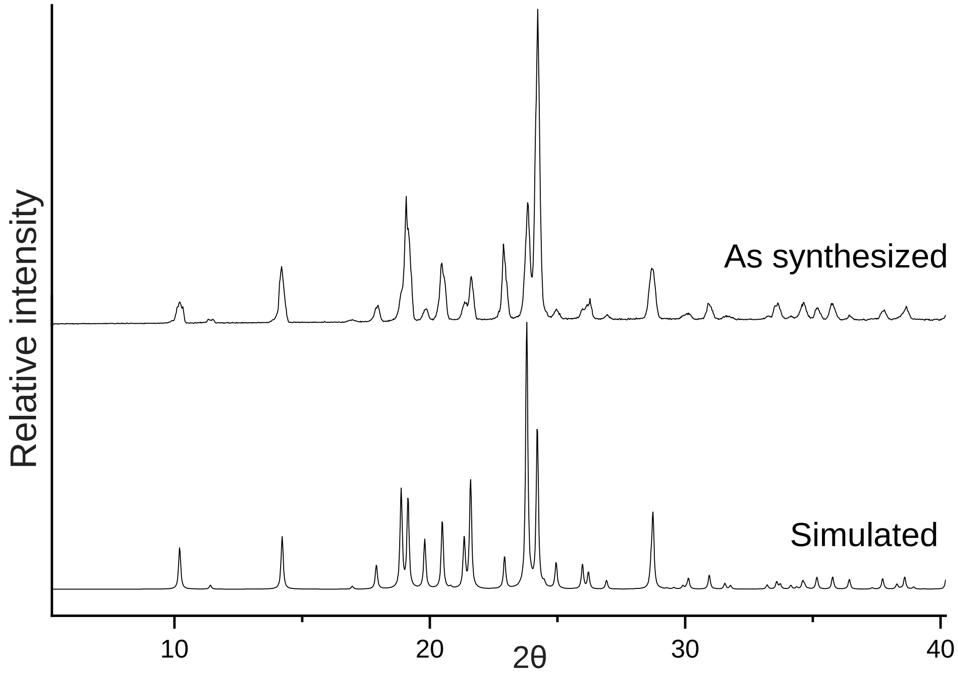

Figure S11. Comparison of the simulated pattern from single-crystal X-ray structure of **4-BBU** with the XRPD pattern of the bulk crystals obtained from MeOH/water.

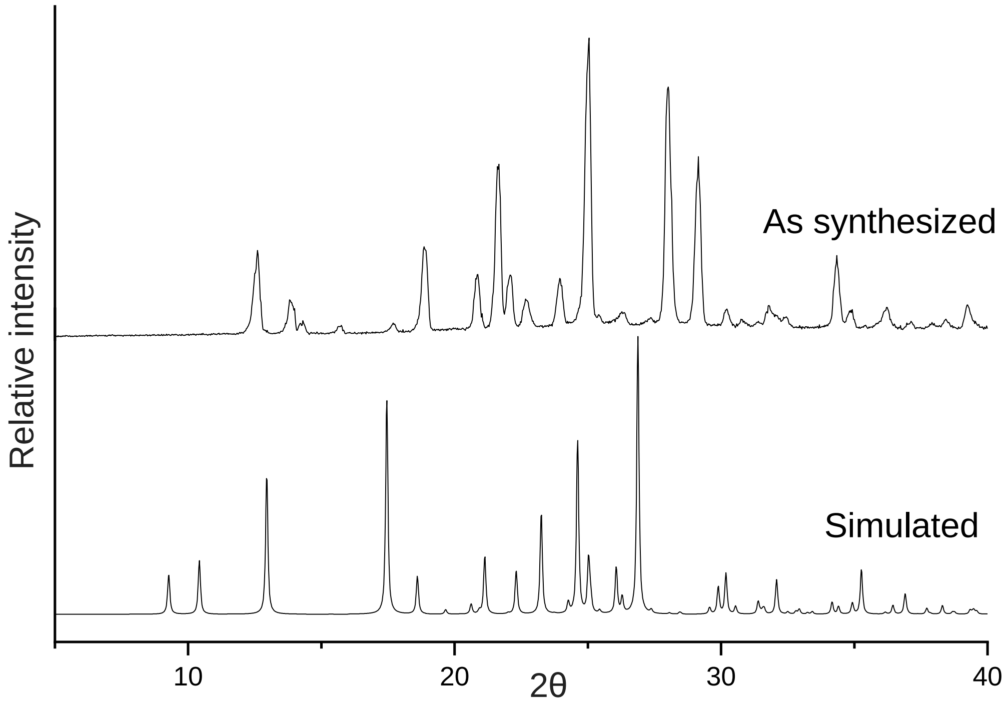

Figure S12. Comparison of the simulated pattern from single-crystal X-ray structure of **4**: with the XRPD pattern of bulk crystals obtained from water.

7. Physical properties in the presence of salts

(a)

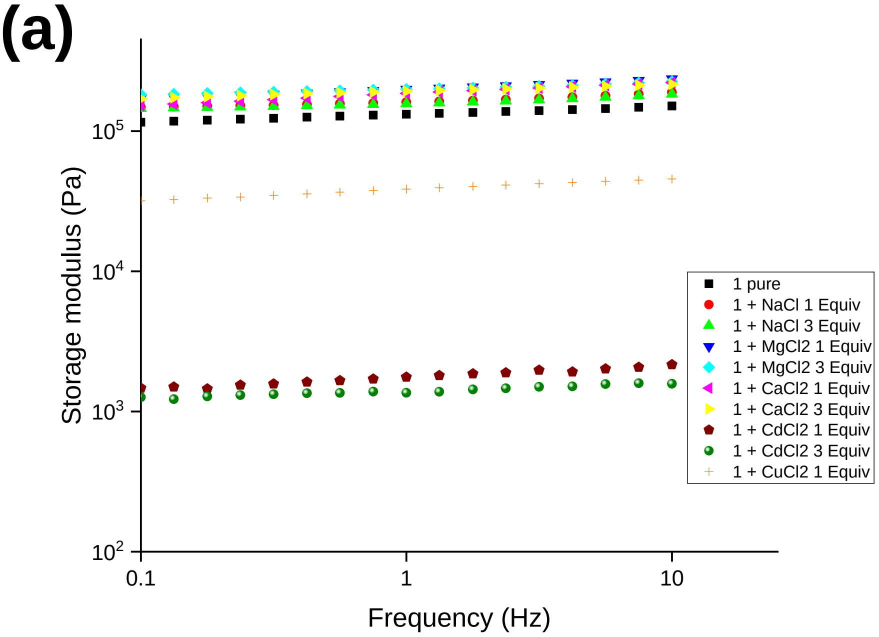

(b)

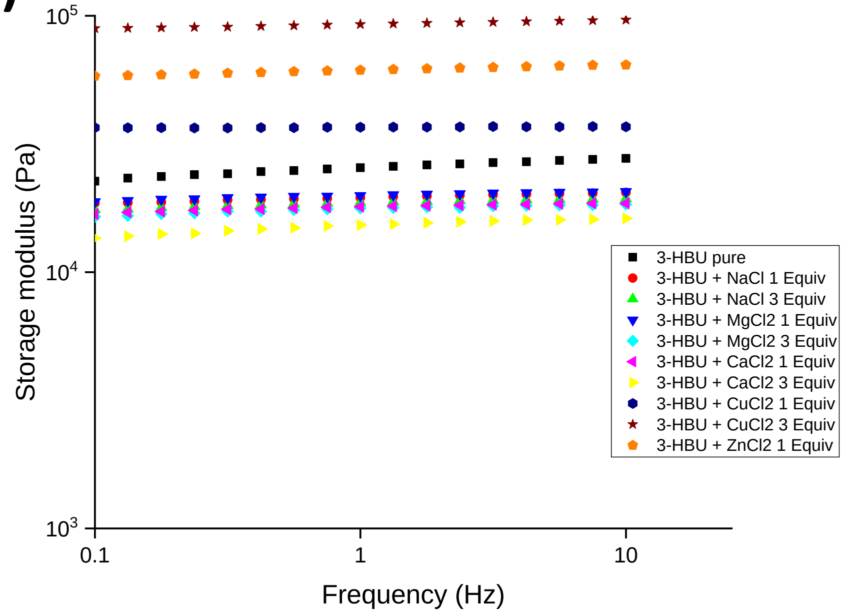

Figure S13. Frequency sweep experiments at 4.0 wt/v% in DMSO/water (1:1, v/v) in the presence of various salts of chlorides at 25.0 °C with a constant strain of 0.02%, (a) gelator **1**, and (b) **3-HBU**.

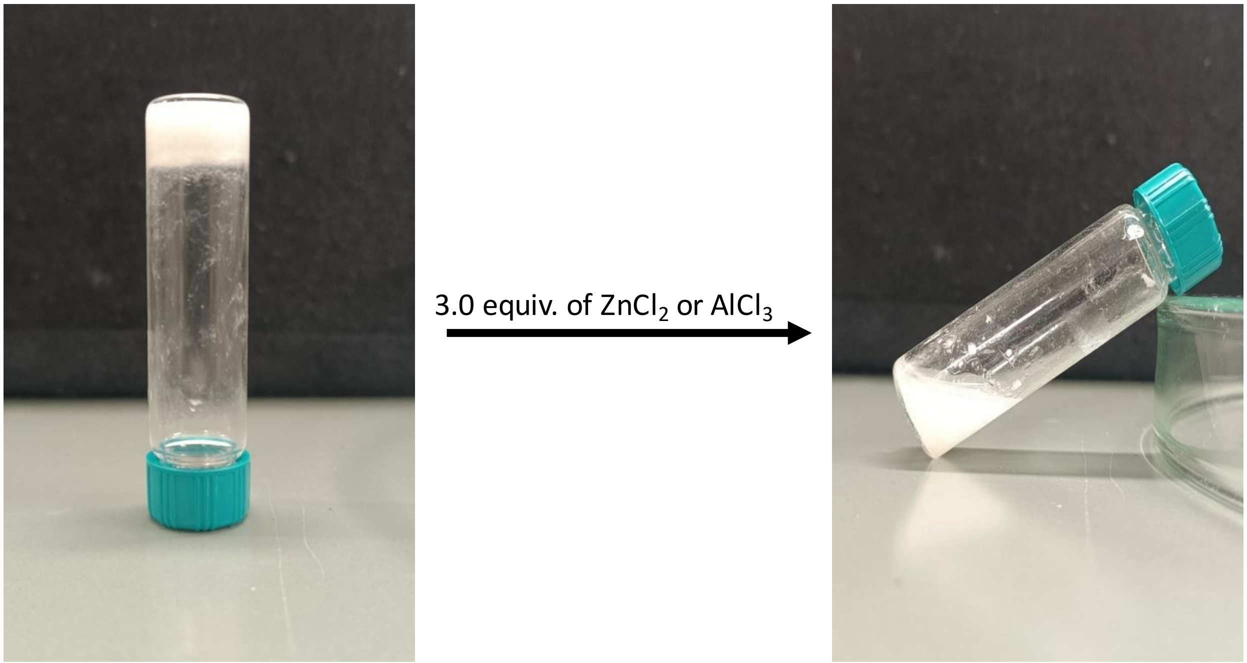

Figure S14. Gel network of **1** collapsing in the presence of 3.0 equiv. of $ZnCl_2$ or $AlCl_3$.

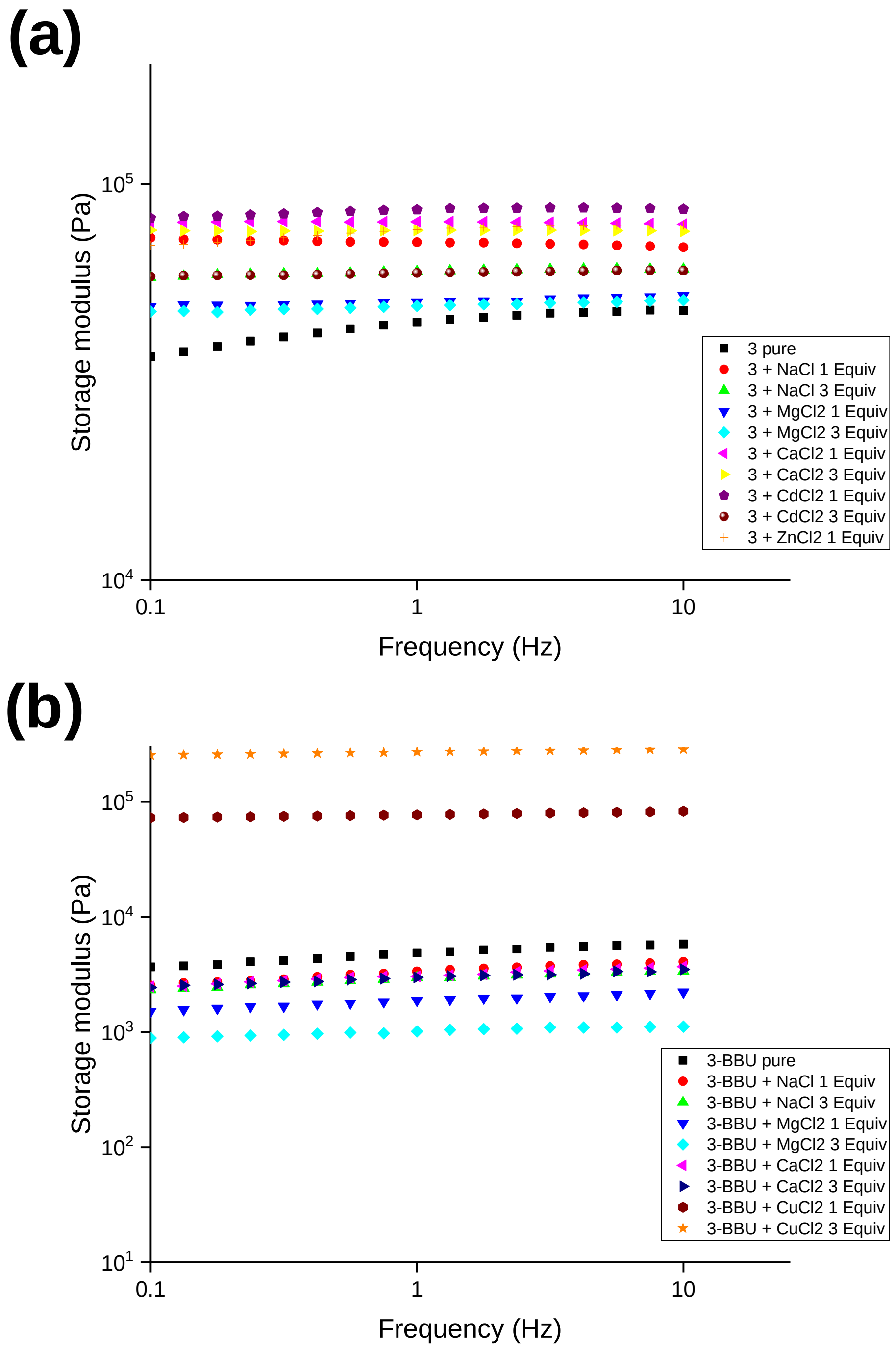

Figure S15. Frequency sweep experiments at 5.0 wt/v% in DMSO/water (1:1, v/v) in the presence of various salts of chlorides at 25.0 °C with a constant strain of 0.02%, (a) gelator **3**, and (b) **3-BBU**.

Table S5: T_{gel} experiments in the presence of salts in DMSO/water (1:1, v/v)

**Salts**	**Equivalence**	T_{gel} **(°C)**			
		**4.0 wt/v%**		**5.0 wt/v%**	
		**3-HBU**	**1**	**3-BBU**	**3**
Ligand pure		93.2	62.9	94.9	61.3
KF	1	91.8	67.8	93.9	62.8
	3	90.3	62.9	88.9	61.1
KCl	1	92.5	67.3	95.0	63.1
	3	90.1	63.1	91.0	59.9
KBr	1	92.1	66.1	91.3	60.3
	3	89.2	63.0	88.3	58.2
KI	1	90.3	66.5	90.4	60.1
	3	89.3	62.0	89.4	59.4
KCN	1	91.1	66.8	88.9	59.4
	3	89.0	62.6	86.4	58.0

Table S6: Stimuli-responsive properties of the parent and modified compounds: comparing the G' values in DMSO/water (1:1, v/v)*

**Salt added**	**Equivalents (equiv.)**	**3-HBU** (4.0 wt/v%)	**1** (4.0 wt/v%)	**3-BBU** (5.0 wt/v%)	**3** (5.0 wt/v%)
KF	1.0	0.8-fold	1.5-fold	0.3-fold	1.1-fold
	3.0	0.7-fold	1.3-fold	0.2-fold	1.1-fold
KCl	1.0	0.8-fold	1.9-fold	0.6-fold	1.6-fold
	3.0	0.2-fold	1.3-fold	0.5-fold	2.5-fold
KBr	1.0	0.7-fold	1.5-fold	0.5-fold	1.1-fold
	3.0	0.5-fold	1.4-fold	0.4-fold	1.1-fold
KI	1.0	0.8-fold	1.9-fold	0.3-fold	1.3-fold
	3.0	0.4-fold	1.3-fold	0.3-fold	1.6-fold
KCN	1.0	0.8-fold	2.0-fold	0.8-fold	1.4-fold
	3.0	0.4-fold	1.4-fold	0.7-fold	1.3-fold
NaCl	1.0	0.8-fold	1.2-fold	0.7-fold	1.4-fold
	3.0	0.7-fold	1.2-fold	0.6-fold	1.3-fold
$MgCl_2$	1.0	0.7-fold	1.6-fold	0.4-fold	1.1-fold
	3.0	0.7-fold	1.5-fold	0.2-fold	1.1-fold
$CaCl_2$	1.0	0.7-fold	1.5-fold	0.6-fold	1.6-fold
	3.0	0.6-fold	1.4-fold	0.6-fold	1.6-fold
$CuCl_2$	1.0	1.3-fold	0.3-fold	14.1-fold	---
	3.0	3.5-fold	---	44.5-fold	---
$ZnCl_2$	1.0	2.4-fold	---	---	1.6-fold
	3.0	---	---	---	---
$CdCl_2$	1.0	---	0.01-fold	---	1.6-fold
	3.0	---	0.01-fold	---	1.2-fold
$AlCl_3$	1.0	---	---	---	---
	3.0	---	---	---	---

*The mechanical strength is compared with the corresponding wt/v% of the pure gelator in the absence of salts.

Table S7: Stimuli-responsive properties of the modified compounds **1**, **3** and **4**: comparing the G’ values in water*

Salt added	Equivalents (equiv.)	Gelator 1		Gelator 3		Gelator 4	
		**2.5 wt%**	**1.5 wt%**	**3.5 wt%**	**2.5 wt%**	**7.0 wt%**	**5.0 wt%**
KF	1.0	1.6-fold	4.5-fold	1.9-fold	4.6-fold	---	---
	3.0	1.7-fold	5.3-fold	1.1-fold	5.2-fold	---	---
KCl	1.0	1.4-fold	6.1-fold	2.9-fold	2.9-fold	---	---
	3.0	1.4-fold	6.5-fold	1.9-fold	3.9-fold	1.1-fold	1.1-fold
KBr	1.0	2.0-fold	1.5-fold	2.0-fold	2.7-fold	---	---
	3.0	2.0-fold	1.3-fold	2.1-fold	4.6-fold	---	---
KI	1.0	1.5-fold	1.2-fold	1.1-fold	4.6-fold	---	---
	3.0	1.2-fold	1.4-fold	1.6-fold	1.2-fold	---	---
KCN	1.0	2.1-fold	1.8-fold	1.3-fold	1.1-fold	---	---
	3.0	1.9-fold	1.9-fold	1.3-fold	1.3-fold	---	---
NaCl	1.0	1.1-fold	3.4-fold	1.4-fold	1.9-fold	---	---
	3.0	1.2-fold	3.1-fold	1.8-fold	3.3-fold	1.4-fold	1.0-fold
$MgCl_2$	1.0	1.2-fold	7.6-fold	1.5-fold	2.4-fold	---	---
	3.0	1.2-fold	5.8-fold	1.7-fold	3.6-fold	1.9-fold	1.7-fold
$CaCl_2$	1.0	1.1-fold	3.6-fold	1.9-fold	2.6-fold	---	---
	3.0	1.1-fold	5.0-fold	1.2-fold	3.6-fold	2.9-fold	2.7-fold
$CuCl_2$	1.0	1.1-fold	1.1-fold	---	2.1-fold	---	---
	3.0	---	---	---	---	---	---
$ZnCl_2$	1.0	1.8-fold	1.1-fold	---	1.5-fold	---	---
	3.0	---	---	---	---	---	---
$CdCl_2$	1.0	1.3-fold	1.1-fold	---	2.3-fold	---	---
	3.0	---	---	---	---	---	---
$AlCl_3$	1.0	1.1-fold	---	1.0-fold	---	---	---
	3.0	---	---	---	---	---	---
$Mg(NO_3)_2$	1.0	1.2-fold	1.3-fold	1.6-fold	1.2-fold	---	---
	3.0	1.2-fold	1.2-fold	1.4-fold	1.8-fold	---	---
$Ca(NO_3)_2$	1.0	1.5-fold	1.4-fold	1.5-fold	2.1-fold	---	---
	3.0	1.5-fold	1.2-fold	1.7-fold	2.5-fold	---	---
$MgSO_4$	1.0	1.6-fold	1.6-fold	1.8-fold	2.2-fold	---	---
	3.0	1.6-fold	1.3-fold	2.0-fold	1.9-fold	---	---
$CsCl_2$	1.0	1.5-fold	1.8-fold	1.9-fold	1.1-fold	---	---
	3.0	1.6-fold	1.3-fold	1.3-fold	1.7-fold	---	---
$SrCl_2$	1.0	1.1-fold	1.6-fold	1.4-fold	2.2-fold	---	---
	3.0	1.3-fold	1.5-fold	1.6-fold	2.3-fold	---	---
$BaCl_2$	1.0	1.6-fold	1.4-fold	1.6-fold	3.8-fold	---	---
	3.0	1.5-fold	1.6-fold	1.9-fold	1.8-fold	---	---

*The mechanical strength is compared with the corresponding wt% of the pure gelator in the absence of salts.

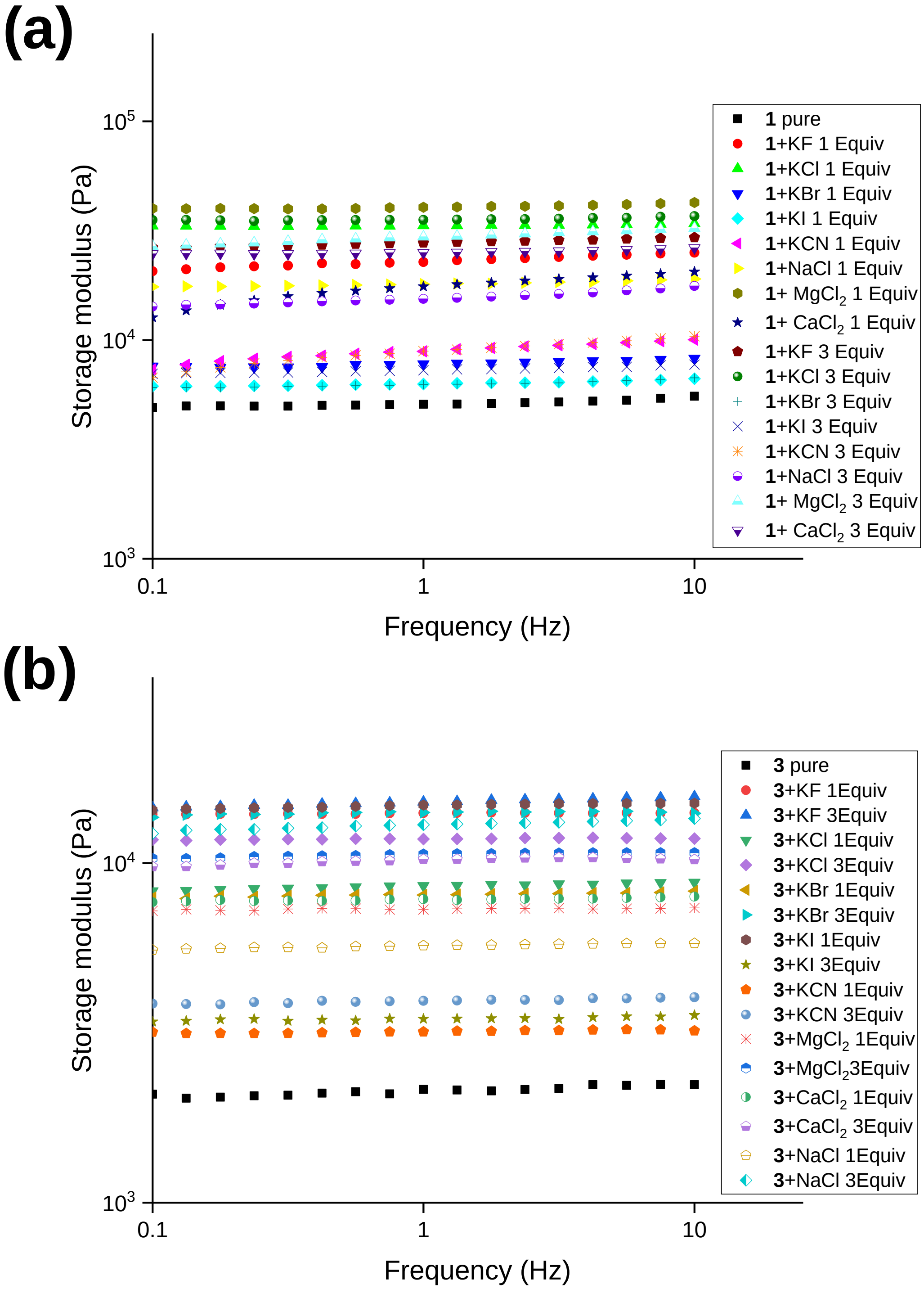

Figure S16. Frequency sweep experiments at MGC in water in presence of salts at 25.0 °C with a constant strain of 0.02%, (a) compound **1** at 1.5 wt%, and (b) compound **3** at 2.5 wt%.

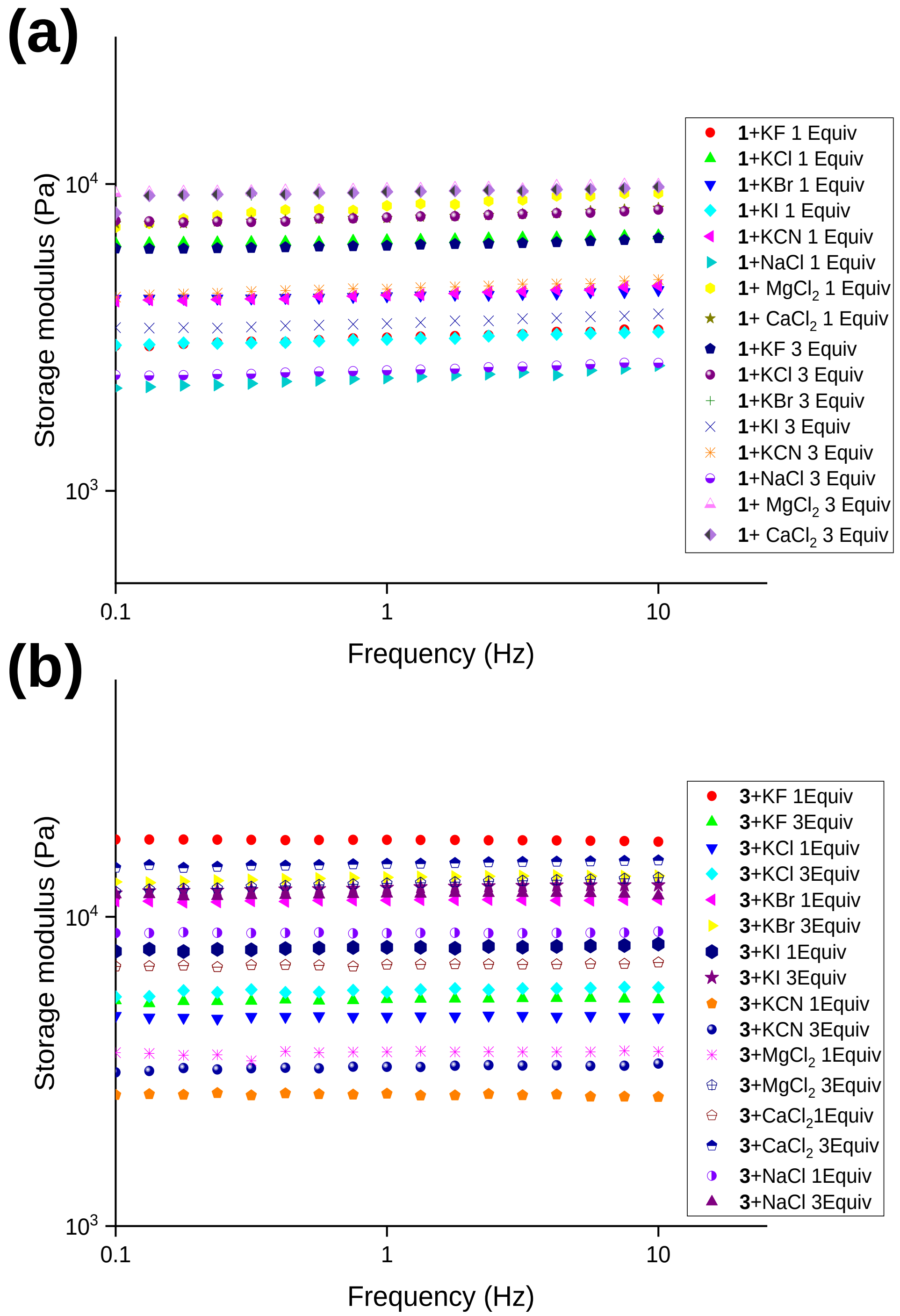

Figure S17. Frequency sweep experiments below MGC in water in presence of salts at 25.0 °C with a constant strain of 0.02%, (a) compound **1** at 1.0 wt%, and (b) compound **3** at 2.0 wt%.

*Physical properties of compound **4** in the presence of salts*: The experiments were first performed at 7.0 wt% of **4** in the presence of chloride salts of potassium, sodium, magnesium and calcium, and the analysis of mechanical strength revealed a 2-3-fold increase of G' in the presence of magnesium chloride and calcium chloride (Figure S18a).The experiments were then performed at 5.0 wt/v% of **4** in water in the presence of various salts such as potassium chloride, sodium chloride, magnesium chloride and calcium chloride. At 1.0 equivalent of the salts, no gel was obtained, but on increasing the salt concentration to 3.0 equivalents, gelation was observed for **4** with all these salts. The mechanical strength of these gels was evaluated by frequency sweep experiments and was compared with the partial gel obtained with the pure compound **4** at 5.0 wt/v%. The comparison of the rheological data revealed that the mechanical strength of **4** was similar to the KCl mixture, but 2-3-fold increase in G' was recorded for the gels with magnesium chloride and calcium chloride (Figure S18b).

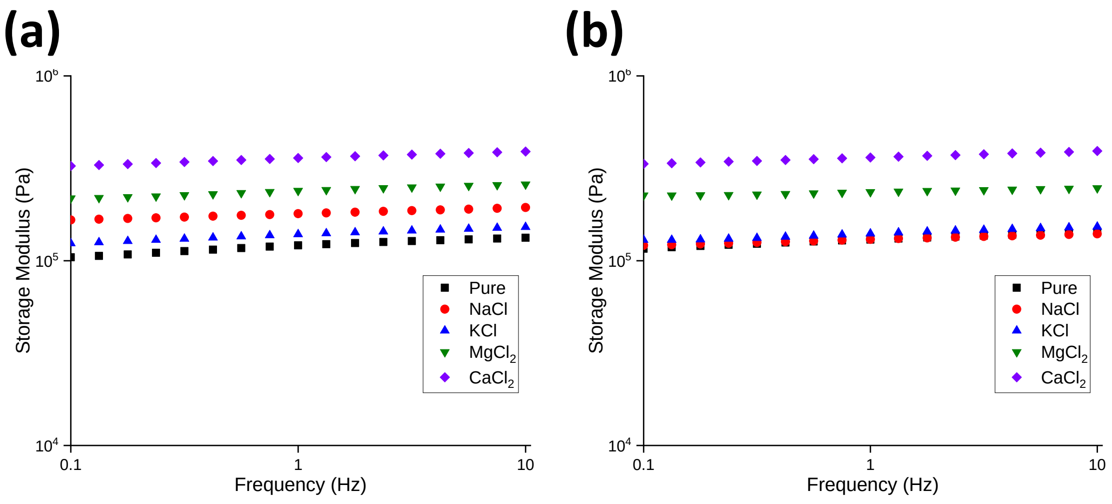

Figure S18. Frequency sweep experiments with compound **4** in the presence of 3.0 equivalents of the salts in water at 25.0 °C with a constant strain of 0.02%, (a) 7.0 wt%, and (b) 5.0 wt%.

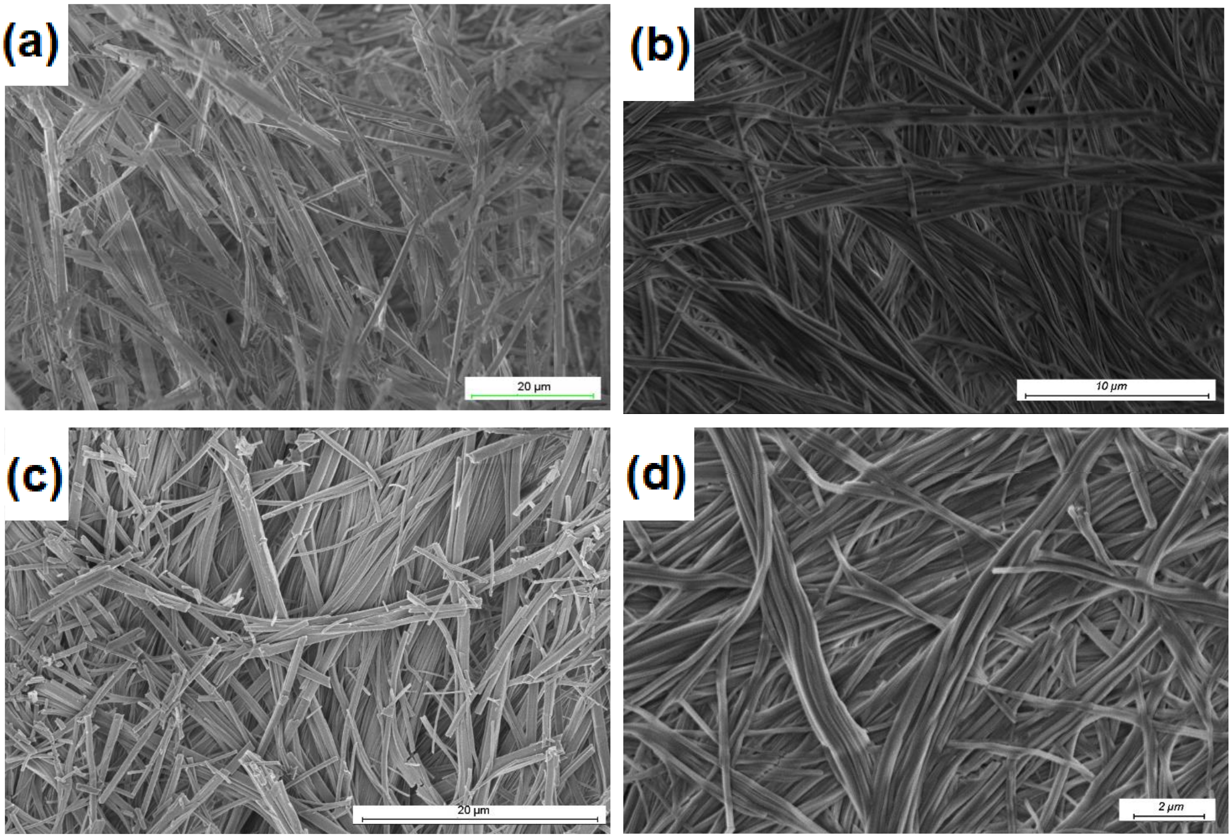

Figure S19. SEM images of xerogels of **1** obtained from pure water at 1.0 wt% (below MGC), (a) partial gel, and in the presence of 3.0 equiv. of (b) potassium fluoride, (c) magnesium chloride, and (d) calcium chloride.

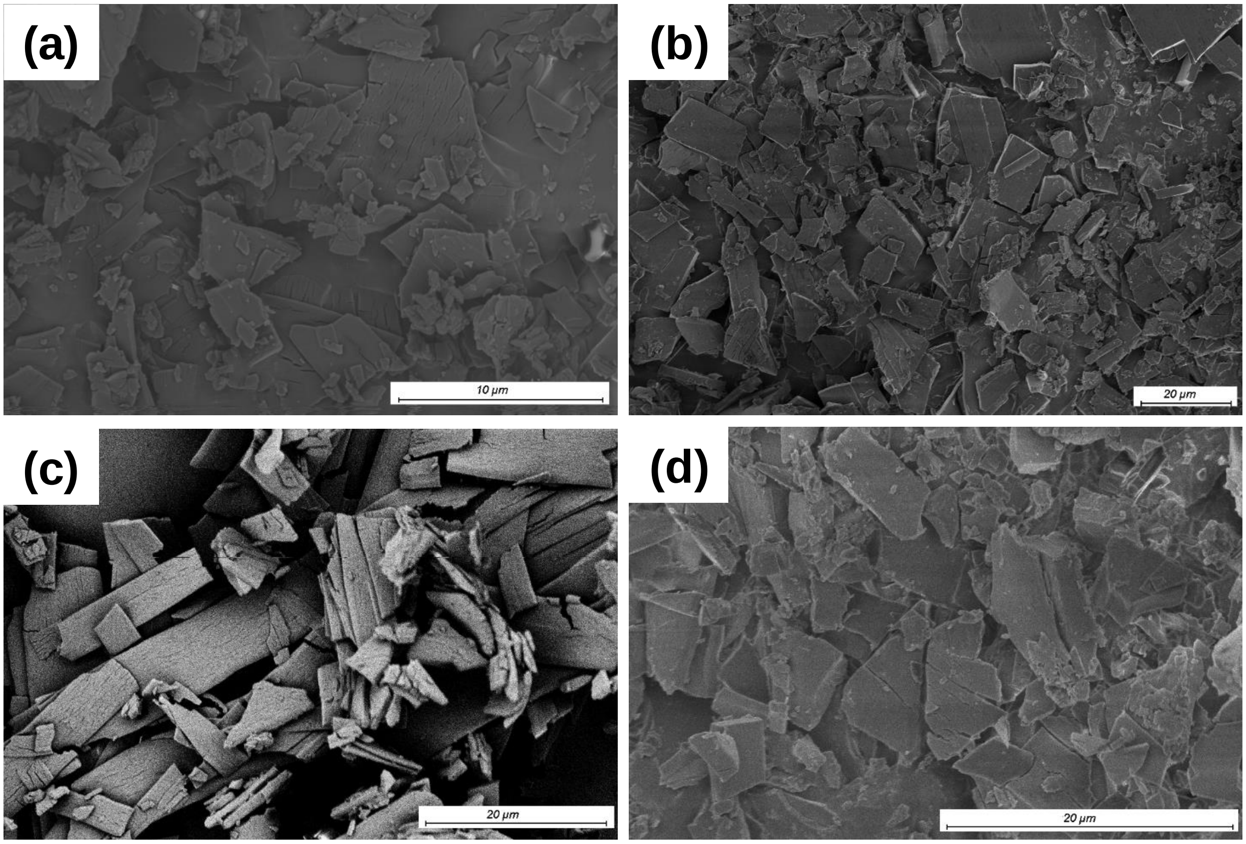

Figure S20. SEM images of xerogels of **3** obtained from pure water at 2.0 wt% (below MGC), (a) partial gel, and in the presence of 3.0 equiv. of (b) potassium fluoride, (c) magnesium chloride, and (d) calcium chloride.

8. NMR spectra

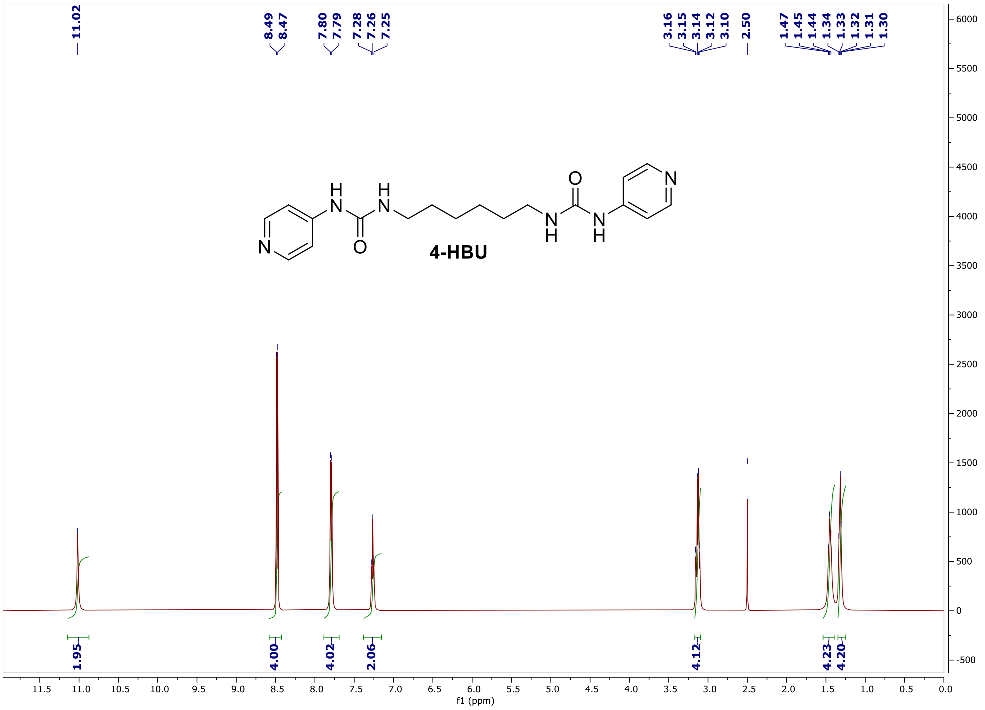

Figure S21. ^{1}H NMR spectrum of compound **4-HBU**.

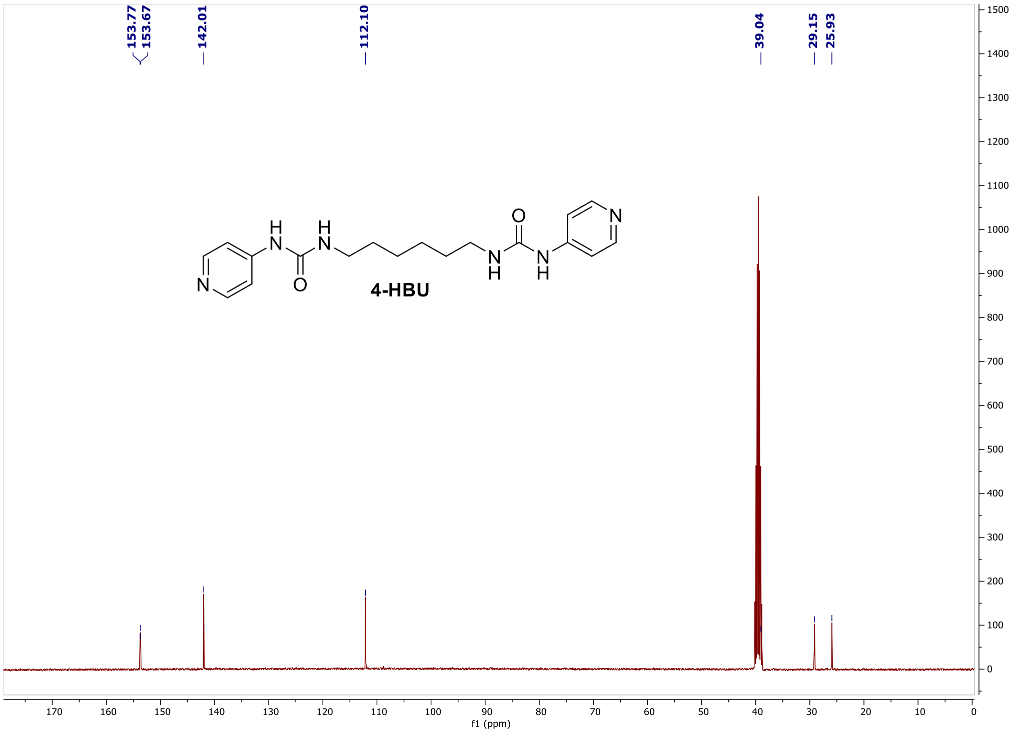

Figure S22. ^{13}C NMR spectrum of compound **4-HBU**.

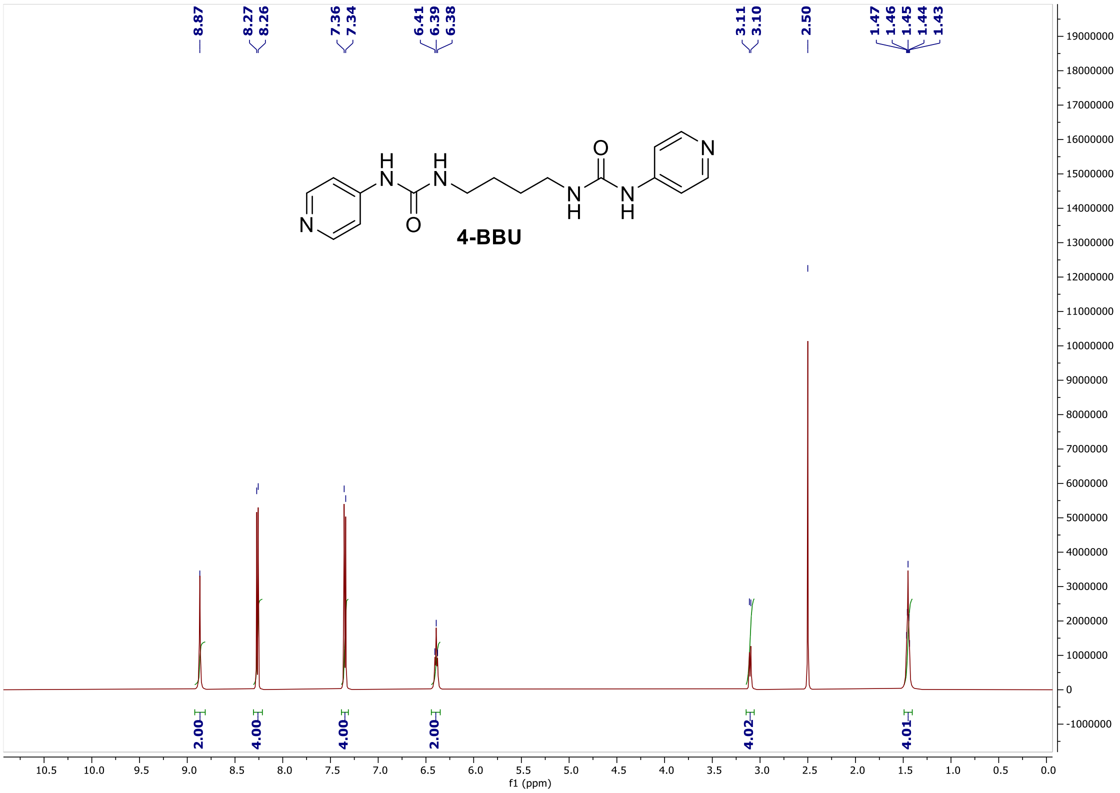

Figure S23. ^{1}H NMR spectrum of compound **4-BBU**.

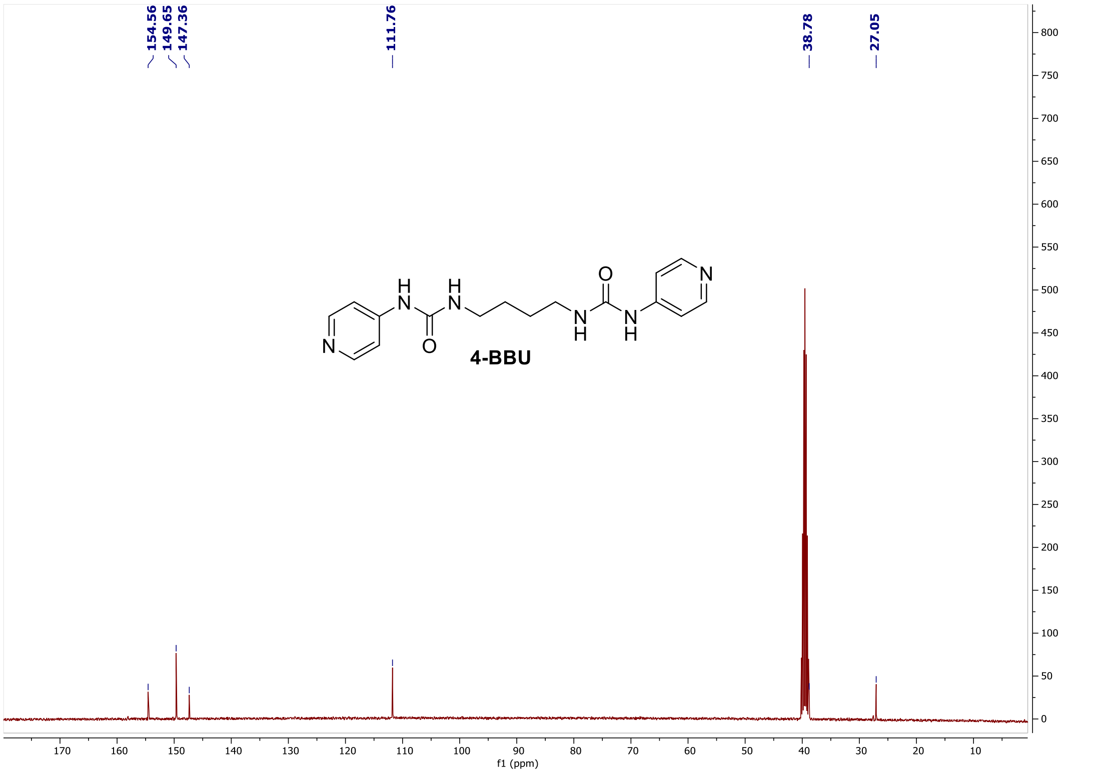

Figure S24. ^{13}C NMR spectrum of compound **4-BBU**.

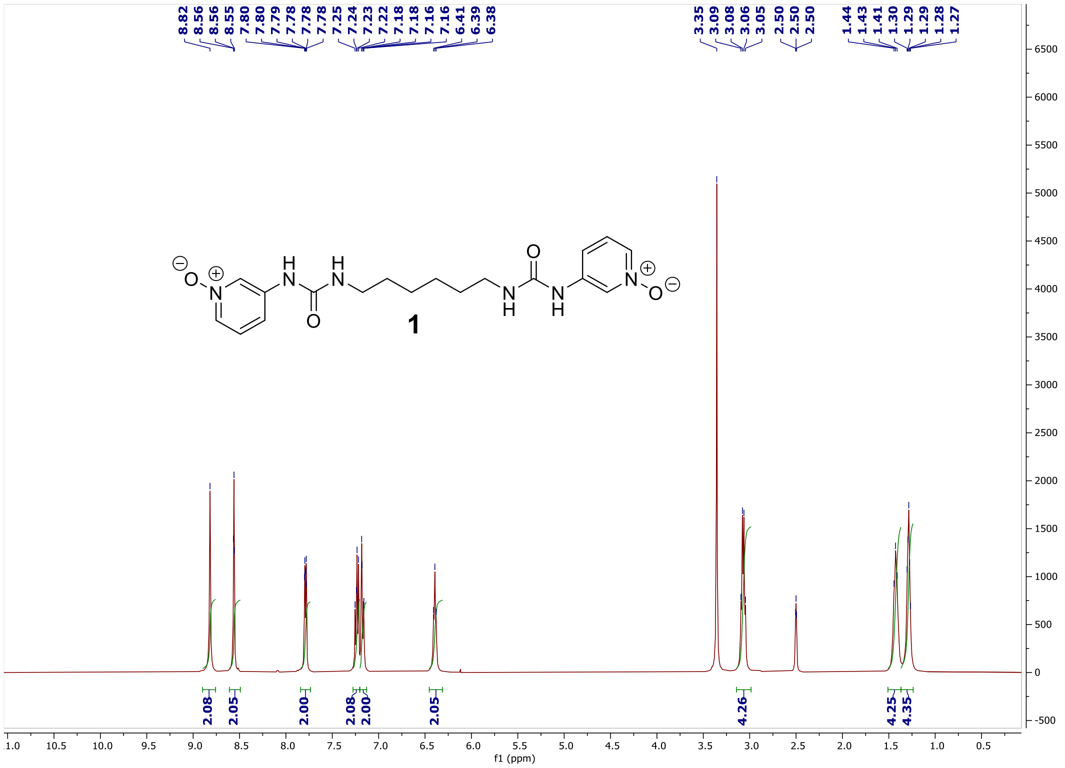

Figure S25. ^{1}H NMR spectrum of compound **1**.

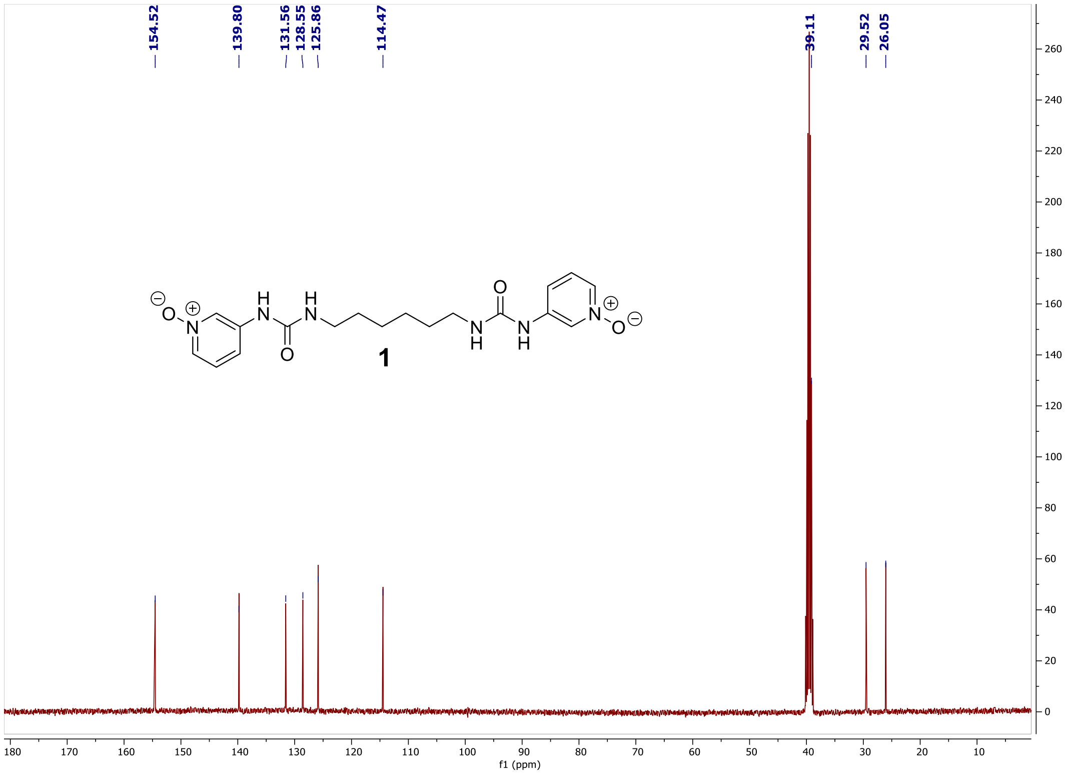

Figure S26. ^{13}C NMR spectrum of compound **1**.

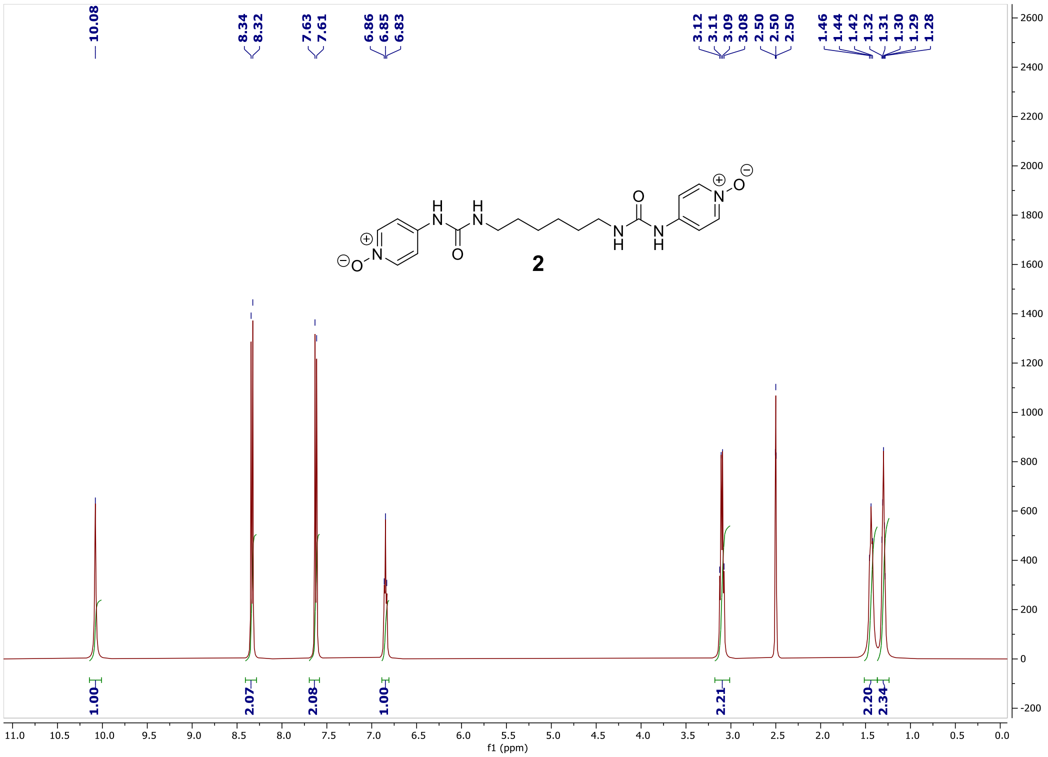

Figure S27. ^{1}H NMR spectrum of compound **2**.

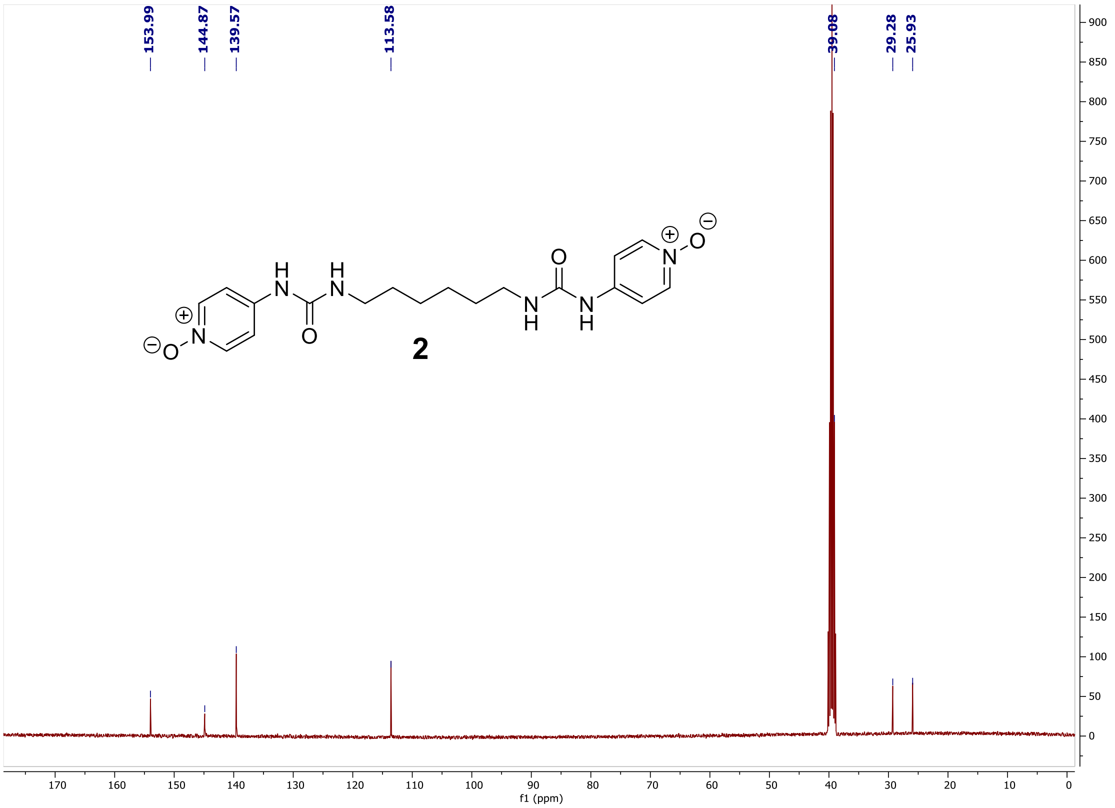

Figure S28. ^{13}C NMR spectrum of compound **2**.

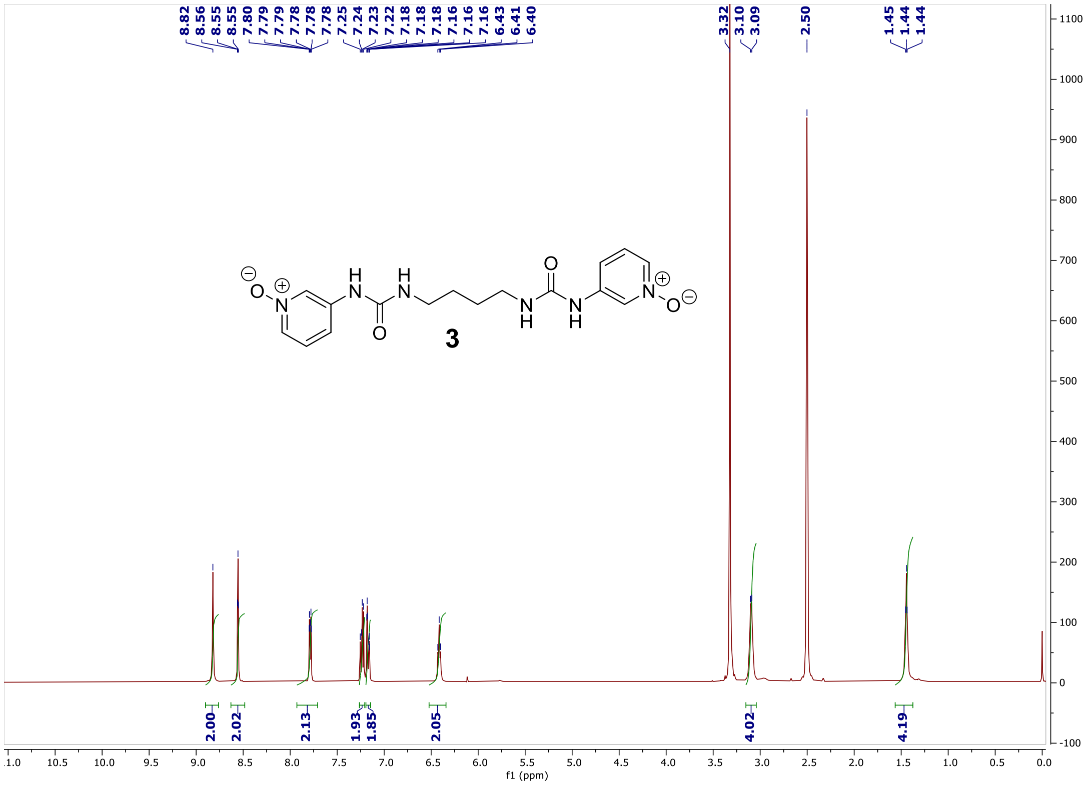

Figure S29. ^{1}H NMR spectrum of compound **3**.

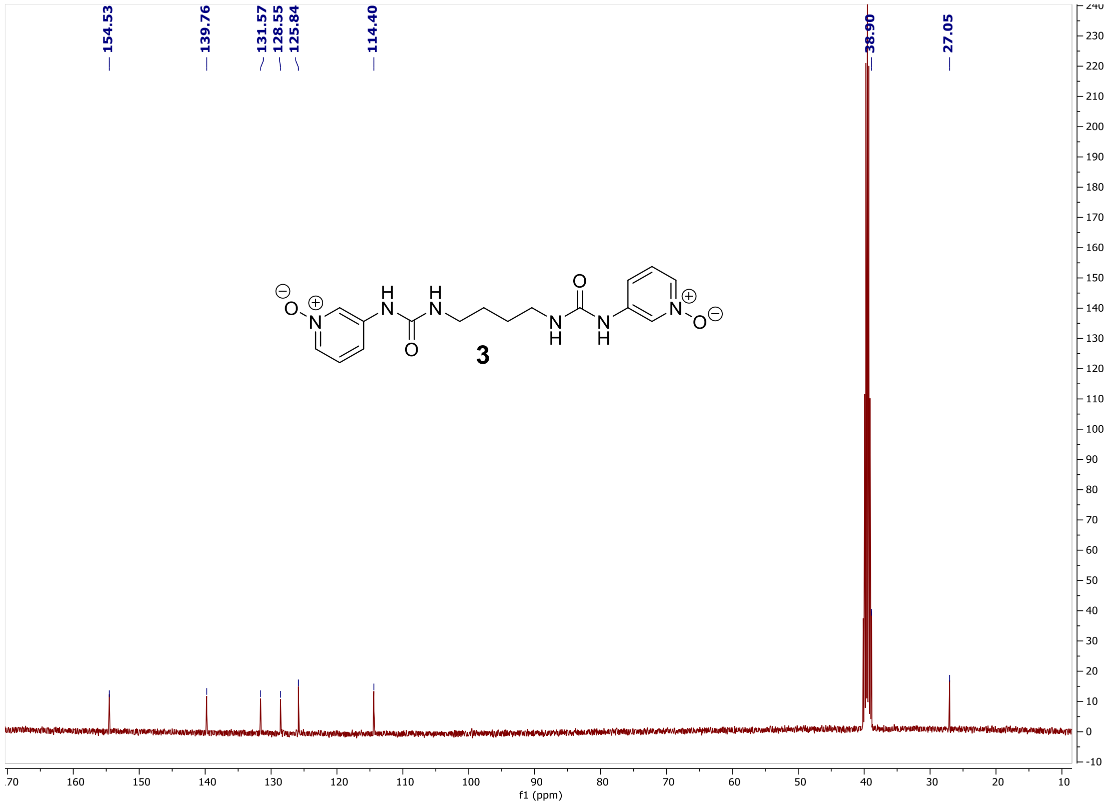

Figure S30. ^{13}C NMR spectrum of compound **3**.

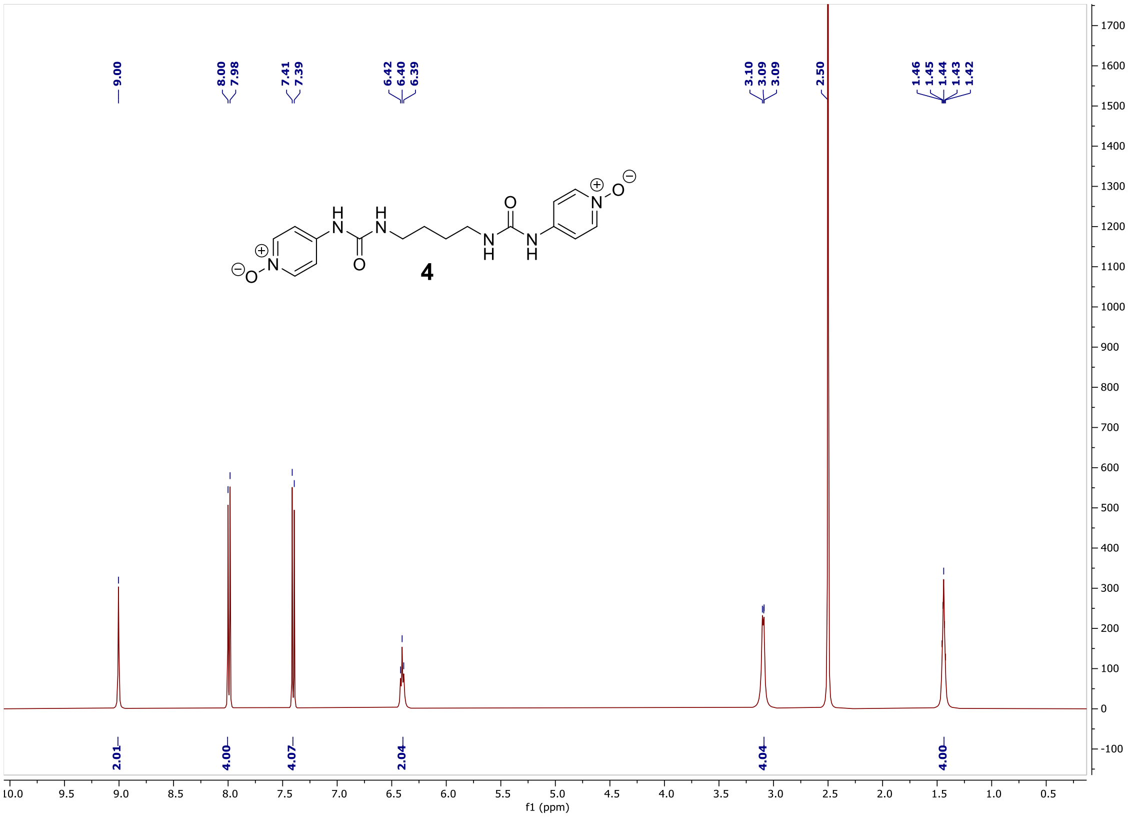

Figure S31. ^{1}H NMR spectrum of compound **4**.

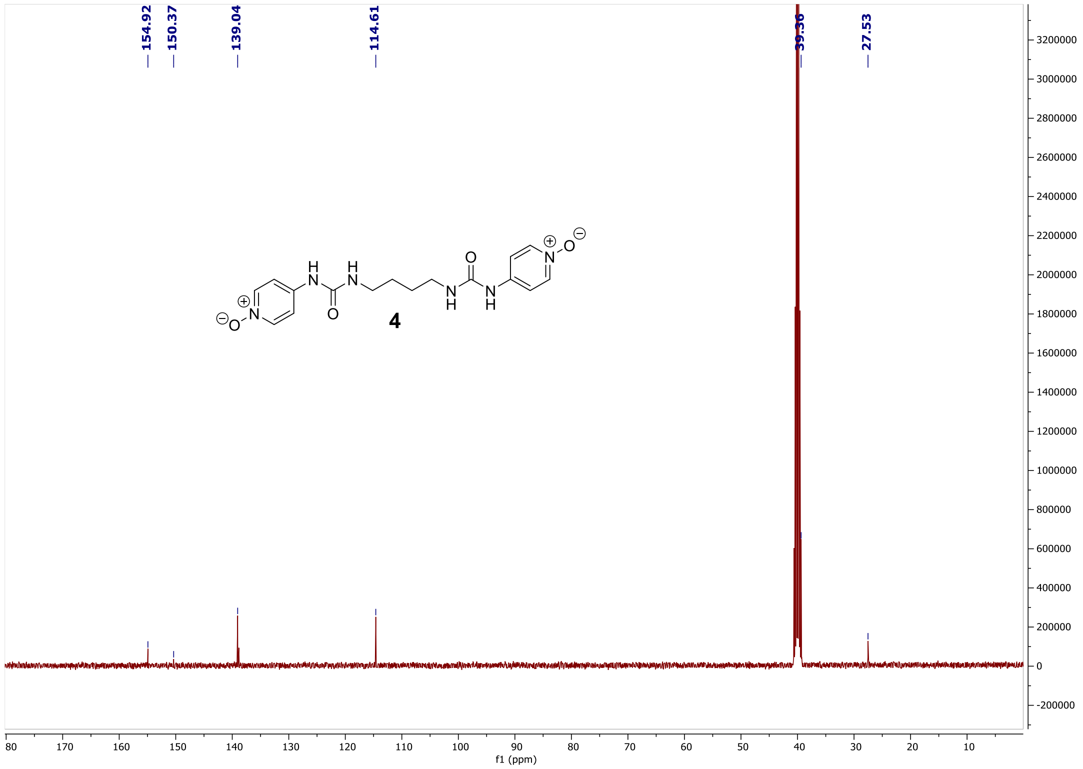

Figure S30. ^{13}C NMR spectrum of compound **4**.
